# Green Synthesis of Silver Nanoparticles Using Plant Extracts: A Comprehensive Review of Physicochemical Properties and Multifunctional Applications

**DOI:** 10.3390/ijms26136222

**Published:** 2025-06-27

**Authors:** Furkan Eker, Emir Akdaşçi, Hatice Duman, Mikhael Bechelany, Sercan Karav

**Affiliations:** 1 Department of Molecular Biology and Genetics, Çanakkale Onsekiz Mart University, Çanakkale 17100, Türkiye; furkan.eker@stu.comu.edu.tr (F.E.); emirakdasci@stu.comu.edu.tr (E.A.); hatice.duman@comu.edu.tr (H.D.); 2 European Institute for Membranes (IEM), UMR-5635, University Montpellier, ENSCM, CNRS, Place Eugène Bataillon, CEDEX 5, F-34095 Montpellier, France; 3 Functional Materials Group, Gulf University for Science and Technology (GUST), Masjid Al Aqsa Street, Mubarak Al-Abdullah 32093, Kuwait

**Keywords:** nanotechnology, nanoparticle synthesis, silver nanoparticles, green synthesis, optimization, plant extracts, antimicrobial activity, agricultural applications, biomedical applications

## Abstract

Green synthesis of silver nanoparticles (AgNPs) using plant extracts has emerged as a sustainable and eco-friendly alternative to conventional physical and chemical methods. This review provides a comprehensive overview of plant-mediated synthesis routes, emphasizing the influence of phytochemicals on nanoparticle formation, morphology, and stability. The physicochemical properties of AgNPs, such as size, shape, and surface characteristics, are critically examined in relation to synthesis parameters, summarizing the plant species employed and associated reaction conditions. The wide-ranging applications of plant-based AgNPs are explored, including antimicrobial, agricultural, environmental, industrial, and biomedical uses, such as drug delivery and wound healing. The section is supported with recent application-specific studies to their corresponding nanoparticle properties, highlighting the relationship between structure and function. Finally, this review discusses current challenges, particularly potential toxicity considerations, and outlines future perspectives for standardization, mechanistic understanding, and translational potential in wide-ranging applications.

## 1. Introduction

Nanoparticles (NPs) have significant applicability in various applications, including antimicrobial research, agriculture, biosensor development, drug delivery systems, and biomedicine [1]. Among these, different types of NPs are distinguished by their unique physicochemical properties. Metal-based NPs, particularly silver NPs (AgNPs), are among the most widely used NPs in current nanotechnological applications. The synthesis of AgNPs dates back over 100 years, with their stabilization using proteins reported a few decades later [2]. The green synthesis approach, on the other hand, was established in the beginning of the 2000s, followed by an expansion in terms of principles in 2012 [3]. The NP production with green synthesis approach quickly gained popularity due to its eco-friendly nature and ability to produce NPs with controlled sizes and shapes, ensuring their stability and bioavailability. Green synthesis of AgNPs can be mediated with various sources, including bacteria, yeast, various algal species, fungal and plant extracts. Among the various natural sources for the synthesis of metal-based NPs, plant extracts offer distinct advantages, especially for the synthesis of AgNPs. Plant-based synthesis offers a cost-effective and efficient approach for the rapid synthesis of highly stable AgNPs, with the added advantage of shorter incubation times compared to other green synthesis methods, such as those involving fungi and bacteria [4]. Moreover, the diversity of phytochemicals in plant extracts allows for control over the physicochemical properties of NPs, offering significant potential for large-scale industrial applications as a scalable method.

**Figure 1:**
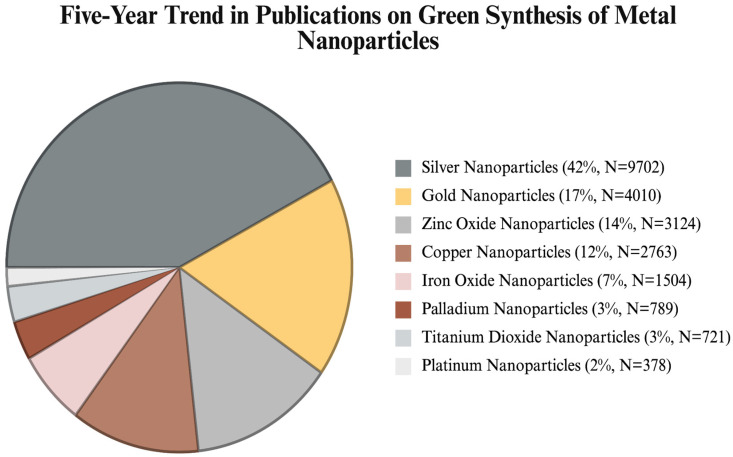
Five-year trend in publications on green synthesis of nanoparticles (Topic Search, Web of Science Core Collection). Data retrieved from the Web of Science Core Collection using “Topic” search for “green synthesis of [nanoparticle type]”; publication counts (N) represent the number of articles indexed in the last 5 years, from 2021 to 2025 (Accessed on 12 May 2025).

Due to their notable antibacterial activity, AgNPs are used in wide-ranging applications, specifically biomedical, pharmaceutical, industrial and environmental fields [5,6]. However, a major drawback of AgNP applications is their potential toxicity, which arises from the synthesis methods used. The conventional synthesis of AgNPs suffers from the agglomeration risk of the particles from the physical synthesis and hazardous side effects from the chemical synthesis [7]. As an alternative, green synthesis approaches have been predominantly utilized in metal NP synthesis as an eco-friendly and sustainable alternative in recent years [8]. Using biological sources, such as plants, bacteria, and fungi, as a reducing and stabilizing agent, green synthesis of AgNPs offers an effective solution for the toxicity problem. Among the various types of green synthesis approaches, plant-based synthesis is one of the most explored types of synthesis in the current literature, as a result of the accessible bioactive compounds, simplicity of the extraction methods, and cost-effective processes [9]. Considering the effect of phytochemicals found in plant extracts, such as flavonoids, proteins and polyphenols, in the reduction of silver ions, plant-mediated synthesis of AgNPs offers enhanced biocompatibility that is essential for the biomedical applications [10]. Consequently, AgNPs have emerged as the most extensively studied material in the current literature on the green synthesis of metal NPs (Figure 1).

Considering the published articles over the last few years, AgNPs have been primarily found in the green synthesis-based research among the metal-based NPs. As illustrated in Figure 1, AgNPs comprises 42% of the total publications within the last 5 years regarding the green synthesis of NPs. In particular, AgNPs dominate the green synthesis of NPs, having a document number of approximately 10,000, doubling the number of publications related to gold NPs. This is mostly attributed to the favorable physicochemical properties of AgNPs, mainly due to their immense antimicrobial activity towards a wide spectrum of pathogens. Another reason is the significance of the literature background of AgNPs, which has been widely tested for their efficiency in applications ranging from biomedical to agriculture. Although AgNPs and gold NPs (AuNPs) have gained significant attention with recent publications, titanium dioxide NPs (TiO_2_NPs) and platinum NPs (PtNPs) are comparatively less explored in recent years. For instance, AuNPs and AgNPs are known for their strong optical properties due to their surface plasmon resonance (SPR), making them promising candidates for photocatalytic and sensor applications [11,12]. In contrast, TiO_2_NPs exhibit a lower light absorption range and catalytic activity, which limits their potential in photocatalytic applications [13]. Similarly, the production of PtNPs is extremely costly, and their stability and aggregation are difficult to manage, making them harder to manufacture and study [14]. Such limitations contribute to the preference for AgNPs and AuNPs in research and application, as they offer more accessible, stable, and functionally tunable alternatives.

This review provides a comprehensive evaluation of plant-based green synthesis of AgNPs, involving the latest findings from diverse application areas, from biomedical to agricultural sectors. We have constructed two tables to highlight the recently used plant sources in the green synthesis and to provide a representative overview of recent studies specific to the covered applications. Given the increasing number of publications on the green synthesis, as highlighted in Figure 1, the present paper provides updated information on this topic, focusing on plant-based routes.

## 2. Properties of AgNPs

Variations in the attributes of NPs were caused by differences in their chemical composition, shape, size, and controlled dispersity [15]. Some of these discrepancies are frequently the result of the synthesis process, which is impacted by a number of different circumstances. The manufacture of NPs in the modern day places an emphasis not only on the achievement of nanoscale dimensions, but also on the guarantee that the synthesis process is uncomplicated, economical, and green, and that it can be adapted to specific applications [16].

In the fields of biosensing, photonics, electronics, drug delivery, and antimicrobial treatments, AgNPs are widely recognized for their exceptional optical, electrical, and antibacterial capabilities [17]. The size of AgNPs usually ranges between 1 and 100 nm. A wide range of applications have found extensive use for AgNPs, which are renowned for their exceptional optical, electrical, and antibacterial capabilities. These applications include biosensing [18], photonics [19], electronics [20], and antimicrobial therapies [21], amongst others.

The morphology of AgNPs is characterized by transmission and scanning electron microscopy (TEM, SEM). Measuring the size distribution of AgNPs is feasible with a Zetasizer Nano Series analyzer. An emission scanning electron microscope that is fitted with an EDS apparatus is utilized in order to carry out measurements using energy dispersive X-ray spectroscopy (EDS). The characterization of AgNPs can also be accomplished through the utilization of X-ray photoelectron spectroscopy (XPS), X-ray diffractometry (XRD), Fourier transform infrared spectroscopy (FTIR), and ultraviolet-visible spectroscopy. The Plasmon resonance is demonstrated by the use of UV-Vis spectroscopy in order to validate the creation of AgNPs. Additionally, XRD is utilized for the purpose of determining the crystallinity of a substance. In order to provide an accurate assessment of the electrical conductivity, the volume resistivity is measured using a Loresta-GP MCP-T610 (Mitsubishi Chemical Corporation, Tokyo, Japan) resistivity meter [16].

### 2.1. Physical Properties

A variety of physical characteristics of AgNPs are intrinsically related to their size, shape, and surface morphology. Both their optical behavior and their biological and catalytic activities are significantly impacted by these features, which are also essential in determining their activities. Particle size reduction results in a significant increase in the surface area-to-volume ratio, which in turn improves the particles’ reactivity and their capacity to interact with biological systems. It is vital to have a thorough understanding of these properties in order to tailor AgNPs to specific applications in the fields of environmental technology, food safety, and medicine [22].

Size is a key characteristic that plays a role in determining the majority of the attributes of NPs [23]. “Quantum size effect” is the term that is used to describe the phenomenon that is responsible for the size-dependent features of NPs. Bulk material is characterized by its properties, which are defined by the average of all the quantum forces that are exerted on all of the atoms that contain the material. It is possible to reach the point when the averaging method is no longer effective by reducing the size of the items. Quantum size effect is the term used to describe the manner in which atoms or molecules behave differently when they are grouped together in small clusters as opposed to when they are found in bulk material. Furthermore, the surface energy surplus is also a contributor to particular features of nanoscale objects. This is due to the fact that the specific surface area significantly increases as the particle size decreases [24].

When all of these factors are taken into consideration, it becomes clear that the size of NPs has a significant impact on their physical characteristics, biological impacts, and toxicity. Therefore, before beginning the process of synthesis, it is necessary to give serious consideration to the size of the product [25].

Morones et al. [26] observed that AgNPs, typically 1–10 nm, adhered to target bacteria’s cell membranes and inhibited cell processes such penetration and respiration. Through bacterial DNA, NPs entered the cell and severely damaged internal functions, killing it. They also found that smaller AgNPs release silver ions, enhancing nanosilver’s antibacterial capabilities. Feng et al. found that silver ions’ interaction with thiol proteins disrupted bacterial enzymatic activity [27]. However, Sotiriou et al. argued that smaller nanosilver releases silver ions faster than larger NPs, generating higher toxicity [28].

Spherical, rod-shaped, triangular, cubic, wire-like, and star-shaped AgNPs are some of the shapes that can be produced through the synthesis of AgNPs [29]. There are a variety of qualities that are affected by the form of the AgNPs, including optical, catalytic, and electrical capabilities. As a demonstration, the form and size of the AgNPs considerably improve the interactions that they have with biological systems. Therefore, they are suitable for use in the field of biomedicine, particularly in the areas of drug delivery systems and coatings applications [16].

In recent years, studies have primarily focused on NP size and how size is the main way for NPs to enter the bacterial cell and destroy target cells. More scientists, meanwhile, have entered into study on the form of the NPs and how shape and size interact in the mechanism of antibacterial action of NPs. Pal et al. [22] and Dong et al. [30] have debated the antibacterial activity of nanosilver with various forms. NPs typically take the shape of circles more frequently than not. Since the beginning of their investigation into topics relating to NPs, researchers have discussed how circular-shaped NPs are able to easily penetrate bacterial cells by traveling through protein channels on the plasma membrane. By optimizing and producing circular-shaped AgNPs, Huang et al. [31] were able to develop a treatment that was effective against the plant disease known as *Bipolaris maydis*. Recent research, on the other hand, suggests that triangular AgNPs have a more robust antibacterial action against bacteria at a high atom density than circular AgNPs do. This is due to the presence of a basal plane, which gives triangular AgNPs a more effective antibacterial action against bacteria [22].

In conclusion, due to the fact that the form of AgNPs has an effect on their capabilities, various shapes offer a variety of distinct benefits, including better antibacterial efficiency, catalytic activity, penetrating capability, and optical features. Because of this, selecting the shape of the AgNPs throughout the synthesis process can ultimately improve their use in a wide variety of applications [22,32].

NP stability is an extremely important feature that needs to be controlled and adjusted during the synthesis. The physicochemical properties of the NP, such as their size, shape, and surface charge, can significantly affect how stable they remain after the synthesis process. In return, their long-term functionality and efficiency in various applications are significantly altered. For instance, small-sized particles with irregular morphologies are more prone to aggregation and exhibit reduced stability, whereas morphologies like spherical NPs with average size tend to maintain greater stability [33]. Similarly, the surrounding environment, temperature, and the capping agents used during NP synthesis significantly influence the stability of the particles, highlighting the importance of green synthesis methods. Green synthesis, with its ability to control these parameters through natural agents, plays a key role in enhancing the stability of NPs. Green synthesis utilizes natural biocompounds as both capping and stabilizing agents in the formation of AgNPs. Key metabolites from plant extracts, such as flavonoids and carbohydrates, act as both reducing and capping agents during synthesis, ensuring the stability of the NP products [34]. This not only enables scalable and sustainable production of AgNPs but also provides an opportunity to control the size, shape, and stability by selecting the appropriate plant extract source.

NPs have a number of critical qualities, one of which is the surface charge, which has a substantial impact on the stability of the NPs as well as their interactions with other molecules. By manipulating the surface charge, it is possible to exert precise control over the behavior of NPs in a variety of settings, hence increasing the ability to influence their aggregation, solubility, and reactivity. In accordance with this, the surface charge is also believed to be essential in biomedical applications, where the improved targeting, absorption, and treatment efficiency results from changing surface charges [35]. In addition, certain approaches to modifying the surface charge of nanoparticles, particularly through surface functionalization, can enhance their stability while simultaneously reducing particle aggregation and potential toxicity [36].

At room temperature, the electrical conductivity of bulk silver is among the highest among all metals [37]. This is a result of the atomic structure of silver, which includes the presence of free electrons. Due to the presence of a valence electron in the outermost shell of silver, electrons are able to move freely, which makes it possible for a conductive pathway to be created. Because of this, it is possible to achieve minimal resistance when an electric field is applied, which improves its electrical conductivity and makes it suitable for use in conductive composite formulations with both organic and inorganic sources of material. The same can be said for AgNPs, where the presence of free electrons is an essential component in determining the electrical conductivity of the particles [38].

It is well known that silver has an exceptional thermal conductivity, which is approximately 429 W/mK when the temperature is at room temperature. Due to this feature, silver is able to transport heat with relative ease. Because of their high surface-area-to-volume ratio, AgNPs have an outstanding heat conductivity, and this ratio is maintained even at the nanoscale [39,40]. Because of the small size of the particles, the efficient transfer of heat by phonons and electrons is the primary reason for the high thermal conductivity. This is made possible by the fact that the particles are relatively small. When this occurs, the resistance and dispersion at the grain boundaries are both reduced. As a result of their lower sintering temperatures, AgNPs have higher thermal conductivity, reduced contact resistance, and oxidative resistance, which makes them appropriate for a wide range of technological and industrial applications [41,42].

### 2.2. Chemical Properties

The surface chemistry of AgNPs, the oxidation states of AgNPs, and their ion release capacities are the primary factors that influence the chemical properties of AgNPs. Silver ions, which are capable of being released from the surface of NPs, have the ability to significantly boost the antibacterial characteristics and potential toxicity capabilities of NPs. In addition, the stability, solubility, and cell-interaction capability of NPs can be altered through the process of surface functionalization with a variety of synthetic or biological compounds. The investigation of these chemical features discloses how AgNPs operate in complex environments and how to manage their behavior for applications that are both safer and more effective [43].

Several studies have shown that AgNP’ surface chemistry, oxidation states, and ion release potential affect their antibacterial activity and toxicity. Surface functionalization with stabilizing chemicals considerably impacts solubility, stability, and biological system interaction. According to Tran et al., AgNPs stabilized with sodium dodecyl sulfate (SDS) demonstrated enhanced antibacterial activity, achieving minimum inhibitory concentrations (MICs) below 1 µg/mL due to increased silver ion release from the NP surface [44]. In addition, oleic acid-stabilized AgNPs demonstrated excellent antibacterial activity against *Escherichia coli* (*E. coli*) with MIC values as low as 1 µg/mL, emphasizing the significance of surface coatings in modulating ion release and biological performance.

De Silva et al. claim that silver ions created from AgNPs can produce reactive oxygen species (ROS), therefore causing oxidative stress and cell death [45]. These ions stop bacterial enzymes, particularly those containing thiol groups, from working, hence blocking respiration and DNA replication. The positive surface charge of AgNPs enables electrostatic attachment to negatively charged bacterial membranes, hence enhancing membrane permeability, cell leakage, and lysis. The study further shows that AgNPs’ minuscule size and tailored surface functionalization let them penetrate bacterial cells and directly interact with internal proteins and nucleic acids, hence influencing protein synthesis and generating genotoxicity.

Akter et al. give extensive toxicological data on AgNPs’ physicochemical properties and lethal effects in numerous biological systems [46]. They showed that AgNPs’ cytotoxicity is strongly related to their physicochemical characteristics including particle size, shape, agglomeration, concentration, surface coating, and solubility. The findings underlined how oxidative stress, mitochondrial malfunction, and later apoptotic pathways are mostly caused by AgNPs dissolving into silver ions. Furthermore, it was observed that smaller AgNPs with bigger surface area-to-volume ratios tend to release more ions and produce more ROS, thereby showing more toxicity. The scientists also underlined that surface coatings such as polyvinylpyrrolidone (PVP) or citrate can stabilize the particles, lower aggregation, and control ion release, hence affecting their biological effects.

### 2.3. Optical Properties

Some of the most prominent aspects of AgNPs include their one-of-a-kind optical properties, which are primarily driven by SPR. This phenomenon, which is induced by collective oscillations of conduction electrons in reaction to light, exhibits strong absorption and scattering in the visible spectrum. The outcomes of this phenomena can be seen in the visible spectrum. Because of their optical response, which is particularly sensitive to particle size, shape, and the medium that surrounds them, AgNPs are valuable in colorimetric sensing, imaging, and therapeutic applications. An understanding of these features at a fundamental level is necessary for the design of optical devices and biosensors that are based on AgNP [47].

Because of the unique optical qualities that they possess, AgNPs have been regarded as having an exceptionally high value. The localized surface plasmon resonance (LSPR), which is a phenomenon in which conduction electrons on the surface of the NP oscillate in resonance with incident light, is the primary factor that is responsible for these features. A significant amount of light, often in the visible spectrum, gets absorbed and scattered as a consequence of this phenomena [48]. Consequently, AgNPs are capable of exhibiting colors that can vary depending on their form, size, and the material that surrounds them [49,50]. In addition, LSPR causes an increase in the electromagnetic fields that are located close to the surface of the NP. Because of this, AgNPs are extremely sensitive to changes in their surrounding environment, which makes them more suitable for usage in a variety of applications, such as biological sensing, imaging, and photothermal therapy [51].

## 3. Mechanism and Optimization of Plant-Based Green Synthesis of AgNPs

The green synthesis of AgNPs aims to minimize the use of hazardous chemicals by utilizing bioactive compounds from plant extracts as reducing and stabilizing agents. These extracts can be obtained from several parts of the plant, including leaves, seeds, flowers, stems, barks, roots or even the whole plant [52]. In recent years, a wide variety of plant species have been used in the green synthesis of AgNPs, showing the diversity in the plant-based approaches.

The specific metabolites found in each plant can vary significantly depending on the species, which influence both the efficiency and characteristics of NP formation. These metabolites are mainly secondary plant compounds such as flavonoids, phenolic acids, polyphenols, terpenoids, alkaloids, saponins and tannins. Presence of hydroxyl (-OH) groups in these molecules is associated with the stabilization and reduction of Ag^+^ to Ag^0^, further facilitating the nucleation and growth of AgNPs. Specifically, phenolic compounds and flavonoids are widely reported to act as effective reducing agents due to their electron-donating capabilities [53].

For instance, quercetin, a polyphenolic compound commonly found in fruits, vegetables and beverages like green tea, donates two electrons through its catechol moiety on the B ring to reduce two Ag^+^ ions to form two Ag^0^ NPs [54]. Similarly, gallic acid, a water-soluble triphenolic compound abundant in many plants like green tea, walnut, strawberry, and banana is extensively used in the green synthesis of AgNPs due to its strong reducing ability and effective capping properties that enable both reduction and stabilization of resulting AgNPs [55].

Beyond their reductive role, these metabolites also play a crucial role in the stabilization of resulting AgNPs. Functional groups present in these molecules, such as hydroxyl, carbonyl, and carboxyl moieties, can adsorb onto the NP surface and form a capping layer that prevents aggregation and enhances colloidal stability. In addition, this capping layer may affect the biological function and surface reactivity of the AgNPs produced [56].

However, the stability of some of these compounds, such as polyphenols, may be affected by external factors such as pH, temperature and light. These conditions may lead to their oxidation or degradation over time, which in turn can reduce their capping efficiency and disrupt long-term stability of the NPs [56]. Accordingly, Cruz-Puma et al. stated in their recent study that higher temperatures, above 80 °C, and extreme pH values, below 4 or higher than 8, can degrade polyphenolic compounds and disrupt their ability to reduce and cap Ag^+^ ions effectively during NP formation [57]. This was supported by broader SPR bands and decreased absorbance intensities which indicate low NP stability and increased aggregation. Further, researchers also stated that exposure to light significantly enhanced the synthesis process, as photoactivated polyphenols accelerated Ag^+^ reduction in comparison to dark conditions that limited their activation and reduced their electron-donating capabilities.

In this review, we highlight approximately 30 different plant sources that have been recently utilized in the AgNP synthesis, as given in Table 1. The evaluated plant sources constitute a diverse range of extracts, including leaves, roots, flowers, and fruit peels. Depending on the plant part, the phytochemical profile of the plant varies, greatly influencing the physicochemical properties of AgNPs in addition to synthesis conditions.

Biological activity of green-synthesized AgNPs may arise from the inherent properties of silver core, the bioactivity of capping phytochemicals, or a synergistic combination of both [58]. Many studies in the current literature demonstrate that phytochemicals contained on the NP surface can enhance or modulate the biological activity of AgNPs, particularly antimicrobial and anticancer effects. For example, Yadav et al. conducted a comparative study on the biological activities of AgNPs green synthesized from *Syzygium aromaticum* L., known to be rich in many phytochemicals such as phenolic compounds, monoterpenes and hydrocarbons [59]. These green-synthesized AgNPs were compared to chemically synthesized and glutathione (GSH)-capped AgNPs. Results revealed that green synthesized AgNPs exhibited the highest antioxidant activity of 74.11%, while chemically synthesized and GSH-capped NPs only achieving 46.62% and 58.78%, respectively. Researchers also stated that the green synthesized AgNPs showed superior mosquito larvicidal activity, with the lowest LC_50_ and LC_90_ values of 4.9 ppm and 30.2 ppm, compared to their counterparts. In another perspective, Tavan et al. compared the biological activities of green synthesized AgNPs from *Perilla frutescens* L. with those of the leaf extract alone [60]. Although Perilla frutescens L., rich in bioactive phytochemicals such as rosmarinic acid, rutin, caffeic acid, and ferulic acid, had moderate antioxidant, antibacterial, antifungal and anticancer properties, its effects notably increased when employed for the green synthesis of AgNPs. In particular, resulting AgNPs had strong antioxidant activity, as evidenced by DPPH and FRAP assays, high antibacterial activity against both Gram-negative *E. coli* and Gram-positive *S. aureus* with a MIC of 0.78 mg/mL, along with a fungicidal effectiveness against *C. Albicans* showing MIC of 8 mg/mL. However, leaf extract alone demonstrated low antimicrobial activity against both bacterial strains and the fungus tested. Concerning the anticancer effects, AgNPs reduced the viability of MCF-7 cells to 43.15% at the highest tested concentration of 600 µg/mL, while plant extract alone reduced viability to 46.75%, showing comparable efficiency. These findings collectively highlight the synergistic contribution of phytochemicals not only in NP formation and stabilization but also in the improvement of their biological effectiveness.

**Table 1:** Plant species utilized in the green synthesis of AgNPs and associated synthesis conditions.

Plant Specie	Plant Part	Synthesis Conditions	Nanoparticle Property	Reference
*Cakile maritima*	- Seed	- 3 mL of plant extract mixed with 1 mM silver nitrate (AgNO_3_) solution (40 mL) - 25 °C - 5 h of reaction time - Dark conditions	- Average particle size ranging from 9.45 to 17.15 nm - Spherical shape - Zeta potential of −37.7 mV	[61]
*Cassia occidentalis* L.	- Seed	- 1:9 volume ratio of plant extract (5 mL) to 0.1 M AgNO_3_ solution (45 mL) - Room temperature - 30 min of reaction time - Dark conditions	- Average particle size of 19.78 nm - Spherical and oval-shaped	[62]
*Ziziphora clinopodioides*	- Leaves	- 50 mg of plant extract mixed with 1 mM AgNO_3_ solution (100 mL) - 80 °C - 30 min of reaction time - Drying of NPs at 60 °C for 24 h	- Sizes below 100 nm - Spherical shape	[63]
*Cucurbita* spp.	- Peel	- 1:2 volume ratio of plant extract to 5 mM AgNO_3_ solution - Room temperature - 90 min of reaction time	- Average particle size of 81 nm - Spherical shape - Zeta potential of −9.96 mV	[64]
*Aloe fleurentinorum*	- Whole plant in powdered form	- 1:1 volume ratio of plant extract (10 mL) to 0.01 M AgNO_3_ solution (10 mL) - 60 °C - pH 8 - 2 h of reaction time	- Average particle size of 26.87 nm - Tetrahedral shape	[65]
*Thymus Vulgaris*	- Whole plant	- 1:5 volume ratio of plant extract (5 mL) to 0.1 M AgNO_3_ solution (50 mL) - 70 °C - 1.5 h of reaction time - Overnight drying of NPs at 70 °C	- Average particle size of 44.6 nm - Spherical shape - Zeta potential of −7.46 ± 4.93 mV	[66]
*Trillium govanianum*	- Rhizome	- 1:1 volume ratio of plant extract (50 mL) to 0.1 M AgNO_3_ solution (50 mL) - 30 °C - Dark conditions - Drying of NPs at 60 °C for 24 h	- Average particle size of 38 nm - Nearly spherical and irregular-shaped - Zeta potential of −38.0 mV	[67]
*Glycyrrhiza glabra* Linn	- Root	- Varying concentrations of plant extract (1 to 5 mL) mixed with 2 mM AgNO_3_ solution to reach a final volume of 100 mL - 1 h of reaction time - Dark conditions - Drying of NPs at 45 °C for 12 h	- Z-average particle size of 69.7 nm - Spherical shape - Zeta potential of −40.3 mV	[68]
*Tagetes erecta*	- Flower	- 1:10 volume ratio of plant extract to 1 mM AgNO_3_ solution - 60 °C - 2 h of reaction time	- Average particle size of 72.6 nm - Spherical shape - Zeta potential of +0.719 mV	[69]
*Martynia annua*	- Root	- 1:9 volume ratio of plant extract (10 mL) to 2 mM AgNO_3_ solution (90 mL) - Dark conditions	- Average particle size of 64 nm - Polygonal morphology - Zeta potential of −21.6 mV	[70]
*Aerva lanata*	- Fruits	- 3 mL of plant extract mixed with 10 mM AgNO_3_ solution - 5 h of reaction time - Dark conditions	- Average particle size of 45.05 nm - Spherical shape	[71]
*Morus nigra* L.	- Whole plant in powdered form	- 1:9 volume ratio of plant extract to 3 mM AgNO_3_ solution - 60 °C - 24 h of reaction time	- Particle size of 170.17 ± 12.65 nm - Spherical shape - Zeta potential of −56.6 ± 0.56 mV	[72]
*Paullinia cupana*	- Leaves (collected in different seasons)	- 2 mg/mL of plant extract mixed with 2 mM (340 µg/mL) of AgNO_3_ solution to reach a final volume of 50 mL - 70 °C - 180 min of reaction time	- Average diameter between 39.33 and 126.2 nm - Spherical shape - Zeta potential of −31.8 mV and −34.9 mV for the NPs synthesized from leaves collected in dry and rainy seasons, respectively	[73]
*Clerodendrum serratum*	- Leaves	- 1:9 volume ratio of plant extract (5 mL) to 1 mM AgNO_3_ solution (45 mL) - Varying ratios (2.5, 4.5 and 6.5 mL) of plant extract/AgNO_3_ solution in a total volume of 50 mL, followed by sonication of 10 min each - 1:9 volume ratio of plant extract (5 mL) to 1 mM AgNO_3_ solution (45 mL) prepared by increasing incubation times (1, 2 and 6 h) in dark conditions, followed by sonication of 10 min each	- Particle size of 31.68 nm - Spherical shape - Zeta potential of +12.98 mV	[74]
*Pandanus tectorius*	- Aerial roots	- 1:10 volume ratio of plant extract (10 mL) to 1.0 mM AgNO_3_ solution (100 mL) - 37 °C - 24 h of reaction time	- Particle size less than 30 nm, with 14.1 ± 0.2 nm as the smallest and 29.0 ± 5 nm the highest - Spherical shape	[75]
*Moringa oleifera*	- Leaves	- 1:40 volume ratio of plant extract (2.5 mL) to 1 mM AgNO_3_ solution (100 mL) - Room temperature - Drying of NPs at 55 °C	- Average particle size of 26.19 nm - Spherical shape	[76]
*Neurada procumbens*	- Leaves	- 1:10 volume ratio of plant extract (5 mL) to 1.0 mM AgNO_3_ solution (50 mL) - 60 °C - Approximately 1 h of reaction time - Dark conditions	- Average particle size of 26.19 nm - Spherical shape	[77]
*Kalanchoe fedtschenkoi*	- Leaves	- 50 mg of plant extract mixed with 1 mM of AgNO_3_ solution - 60 °C - 30 min of reaction time - Dark conditions	- Average diameter between 39.9 and 111 nm - Spherical, nanoflower and nano-popcorn shaped - Zeta potential ranging from −7.9 mV to −20.5 mV	[78]
*Hibiscus tiliaceus* L.	- Leaves	- 1:9 volume ratio of plant extract (10 mL) to 1 mM AgNO_3_ solution (90 mL) - 25 ± 2 °C - 30 min of reaction time	- Particle size between 30 and 35 nm - Spherical shape - Zeta potential of −49 mV	[79]
*Sambucus ebulus*	- Leaves	- 1:1 volume ratio of plant extract (100 mL) to 2.0 mM AgNO_3_ solution (100 mL) - 60 °C - 2.5 h of reaction time - Drying of NPs by lyophilization (freeze drying)	- Average particle size of 18.6 nm - Spherical shape	[80]
*Lycium shawii*	- Leaves	- 1:9 volume ratio of plant extract (10 mL) to 0.1 M AgNO_3_ solution (90 mL) - 50 °C - Dark conditions	- Average particle size of 64 nm and 79 nm for NPs from leaf extracts in water (Aq-AgNP) and methanol (MeOH-AgNPs), respectively - Spherical shape - Zeta potential of −29.2 mV for Aq-AgNPs and −22.7 mV for MeOH-AgNPs	[81]
*Acacia raddiana*	- Leaves	- 1:19 volume ratio of plant extract (5 mL) to 5 mM AgNO_3_ solution (95 mL) - 70 °C - 2 h of reaction time - Drying of NPs between 50 and 80 °C	- Particle size between 8 and 41 nm - Spherical and rod-shaped - Zeta potential of −32.2 mV	[82]
*Lallemantia royleana*	- Leaves	- 1:9 volume ratio of plant extract to 1 mM AgNO_3_ solution - Room temperature - 150 min of reaction time - Drying of NPs at room temperature	- Average particle size of 34.47 ± 1.6 nm - Spherical shape - Zeta potential of −24.1 mV	[83]
*Ocimum americanum* *Azadirachta indica* *Allium sativum* *Eucalyptus hybrida* *Zingiber officinale*	- Leaves (Ocimum americanum, Azadirachta indica, Eucalyptus hybrida) - Cloves (Allium sativum) - Rhizome (Zingiber officinale)	- 1:9 volume ratio of plant extract (20 mL) to 1 mM AgNO_3_ solution (180 mL) - Room temperature - 48 h reaction time - Dark conditions	- Particle size ranging from 22.8 to 250 nm for *O. americanum* and 26.6 to 500 nm for *A. indica* - Spherical to irregular shapes for *O. americanum* and *A. indica*	[84]
*Ginkgo biloba* *Cichorium Intybus* *Adiantum Capillus-Veneris* *Rosmarinus Officinalis*	- Whole plants in powdruiered form	- 5:7 volume ratio of 0.01 M AgNO_3_ to plant extract - Room temperature - Drying of NPs at room temperature	- Average particle size ranging from 80 to 100 nm - Spherical shape	[85]
*Anchusa Officinalis*	- Leaves	- 1:4 volume ratio of plant extract (125 mL) to 1 mM AgNO_3_ solution (500 mL) - 22–25 °C - pH 8 - 30 min reaction time - Drying of NPs at 65 °C for 48 h	- Average particle size of 28.5 nm - Spherical shape - Zeta potential of −21.2 mV	[86]
*Lagerstroemia speciosa*	- Ft	- 1:9 volume ratio of plant extract to 1 mM AgNO_3_ solution - 90 °C - 15 min reaction time	- Average particle size of 28.5 nm - Spherical shape - Zeta potential of −21.2 mV	[87]
*Parthenium hysterophorus*	- Flower	- 9:1 volume ratio of plant extract to 1 mM AgNO_3_ solution - 35 °C - pH 7 - 24 h of reaction time - Dark conditions	- Particle size between 25 and 50 nm - Rod- and thread-like shape	[88]
*Pongamia pinnata* L.	- Leaves	- 1:9 volume ratio of plant extract (5 mL) to 2 mM AgNO_3_ solution (45 mL) - Microwave irradiation applied as a 90-s pulse - Room temperature - pH 10 - Dark conditions	- Particle size ranging from 1 to 3.5 nm - Spherical shape - Zeta potential of −38.0 mV	[89]
*Cichorium intybus*	- Leaves	- 1:10 volume ratio of plant extract (5 mL) to 1 mM AgNO_3_ solution (50 mL) - 75–80 °C - 30 min of reaction time	- Average particle size of 79 ± 4.9 nm - Zeta potential of −38.2 ± 5.4 mV	[90]

**Figure 2:**
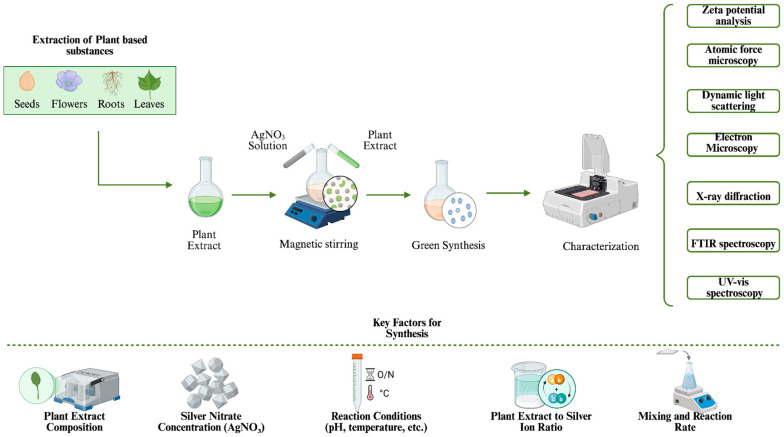
Mechanism and key factors in the plant-based green synthesis of AgNPs [78,92].

The efficiency and characteristics of synthesized AgNPs depend on several factors, such as the choice of plant extract, reaction conditions, and mechanisms of ion reduction and stabilization (Figure 2). Depending on the plant source, the composition of derived polyphenols, flavonoids, and proteins can vary significantly, which in turn can greatly influence the synthesis process. The plant-based synthesis of AgNPs comprises several steps: reduction in the silver ions, nucleation of the reduced silvers and their stabilization. The silver ions, entrapped in the surface of the plant-derived proteins, are reduced with the bioactive compounds in the plant extracts, forming Ag^0^ atoms [91]. These reducing agents can vary from polyphenols and flavonoids to different proteins and organic acids. Following the reduction process, Ag^0^ atoms undergo nucleation, where they aggregate to form small NP seeds. The aggregated Ag^0^ ions are further stabilized and capped with plant biomolecules that serve as capping agents, forming the AgNPs while preventing their aggregation.

Several environmental factors influence the yield, size, morphology, and stability of AgNPs during synthesis. Optimizing these factors is essential to achieving desired physicochemical properties. Variables such as concentration of the plant extract, temperature, pH, AgNO_3_ concentration, and reaction time can greatly influence the physicochemical properties and activity of AgNPs. Key physical characteristics, including size, morphology, and stability, are directly affected by the synthesis process. Notably, the stability of AgNPs is highly affected by the pH and temperature, as their extreme levels negatively influence the NP formation and stability in the long-term. For instance, while higher temperatures can accelerate the formation of AgNPs (with optimum levels typically around 40 °C), lower temperatures can help maintain their long-term stability during storage [93]. Similarly, pH levels around 6 yields highly stable AgNPs, while higher pH levels, such as 10, accelerate the agglomeration of the particles [94]. Therefore, careful control of these parameters is crucial for achieving stable, well-dispersed NPs with desired properties.

Song et al. investigated the impact of temperature on the formation rate of AgNPs using various leaf extracts during synthesis [95]. Their findings revealed that increasing the reaction temperature from 25 °C to 55 °C significantly enhanced the formation rate of AgNPs from 60% to approximately 100%. Additionally, they showed that particle size is strongly influenced by reaction temperature, extract concentration, and AgNO_3_ concentration, with reported sizes ranging from 15 to 500 nm. Another study highlights the variation in AgNP sizes synthesized from different plant extracts and their antibacterial activity [96]. Among the five plant extract sources, AgNPs synthesized from *Solanum tricobatum* and *Ocimum tenuiflorum* exhibited the highest antibacterial activity against *Staphylococcus aureus* (*S. aureus*) and *E. coli*, each producing a 30 mm zone of inhibition at the highest concentration (100 μL), respectively. Notably, despite AgNPs synthesized from *Citrus sinensis* extracts being approximately three times larger (≈65 nm) than those from other sources, they still demonstrated higher antibacterial activity than some of the smaller-sized AgNPs, except against *Klebsiella pneumoniae*. Given that smaller AgNPs generally exhibit greater antibacterial activity, these findings emphasize that the plant extract source plays a crucial role in determining both the size and efficacy of AgNPs. Therefore, the relationship between plant extract composition and AgNP antibacterial properties requires further investigation.

Melkamu et al. demonstrated the effect of AgNO_3_ to plant extract ratio on the absorbance and reduction rate of the AgNPs [97]. A 1:2 ratio resulted in the fastest Ag^+^ reduction and the highest absorbance peak, whereas ratios of 1:1, 1:3, and 1:4 led to lower absorbance peaks and slower reduction reactions. Similar optimal conditions for achieving the highest absorbance peak and the fastest reduction rate were observed at a temperature of 40 °C, an AgNO_3_ concentration of 4 mM, and a pH range of 8–9. Similarly, Manosalva et al. revealed the impact of plant extract concentration, pH and AgNO_3_ concentration on the synthesis of AgNPs using *Galega officinalis* plant extract [98]. In their study, NPs were synthesized from alternating plant extract concentrations (ranging from 10 to 30% *v*/*v*), at different pH values (between 8 and 12) and AgNPs concentrations (from 1 to 5 mM). Results revealed reduction in size distribution as the pH is increased, with smaller sized NPs generated when pH was nearly 11. Also, size distribution of the NPs was determined to be proportional to the AgNO_3_ concentration, while plant extract concentration did not show any significant effects on NP size. They have also stated that NPs had lower zeta potential, smaller than −30 mV, when pH was increased up to 11, AgNO_3_ concentration was high, and plant extract concentration was low. At last, the reaction parameters, including pH in the reaction medium, plant extract concentration and AgNO_3_ concentration, were shown to affect the polydispersity index (PDI) of the NPs, which reflects the uniformity of NP size distribution.

From another perspective Liaqat et al. highlighted the effect of different plant extracts, *Eucalyptus camaldulensis* and *Terminalia arjuna*, in addition to other parameters, including time, temperature, pH and AgNO_3_ concentration, on the optimization of AgNP synthesis [99]. To achieve maximum yield and stability, they have conducted AgNP synthesis at various reaction times, ranging from 15 to 60 min with extracts of *E. camaldulensis*, *T. arjuna* and combinations of these two. The optimum incubation time was found to be 60 min, confirmed by UV-Vis spectra analysis. However, when both plant extracts were utilized, incubation time was reduced to 30 min due to the combination of plant extracts having an enhanced synergistic effect for the reduction of Ag^+^ to Ag^0^, resulting in a shorter reaction time. The optimum reaction time for AgNPs synthesis was recorded as 75 °C, in compared to 25, 50, and 100 °C, indicated by maximum absorbance at a characteristic ʎ_max_ of AgNPs. It was also stated that further increase in temperature (100 °C) led to decrease in the absorbance and shifted the ʎ_max_. Moreover, it was revealed that pH varying from 1 to 5 was not suitable for AgNP synthesis, as it directly influences the size of the NPs, and biomolecules’ capacity to capping and stabilizing by altering their electrical charges. Highly alkaline pH was also unsuitable as it led to undesirable results such as agglomeration of NPs at pH 11. Thus, the neutral pH, 7, was determined as the optimal condition for the synthesis. Considering the AgNO_3_ concentration, 1 mM was found to achieve maximum yield among 0.50, 1.0, 1.5, and 2.0 mM, shown by lower concentrations, 0.50 mM, being insufficient for NP synthesis and higher concentrations, 2 mM, shifting the SPR peaks of AgNPs. In addition, the optimum ratio of *E. camaldulensis*-AgNO_3_ and *T. arjuna*-AgNO_3_ was determined as 3:7 and 4:6, respectively. Authors demonstrated that AgNPs prepared from different plant extracts generated NPs with varying sizes. *E. Camaldulensis* extract, *T. arjuna* extract, and their three different combinations (1:1, 2:1, 1:2) yielded NPs with an average size of 43, 23, 12, 13 and 42 nm, respectively. At last, these combinations of AgNPs had distinct bactericidal efficiency, with the 1:1 combination and *T. arjuna*-extracted AgNPs having the highest zone of inhibition of 16 mm against *Bacillus subtilis,* and 1:2 combination showing the largest zone of inhibition against *S. aureus*.

In a recent study, Ni et al. focused on the optimization of reaction duration, reaction temperature, pH and concentrations of AgNO_3_ and plant extract for the green synthesis of AgNPs from *Ginkgo biloba* leaves [100]. Under the same conditions, synthesis of AgNPs at 90 °C, in comparison to 30, 40, 60 and 80 °C, led to generation of larger amounts of NPs supported by a more intense SPR peak than room temperature. Additionally, researchers observed asymmetric SPR peaks at higher temperatures, indicating the non-homogeneous dispersion of NPs. Analysis on reaction time (30, 40, 60, 75 and 90 min) revealed AgNPs synthesis were completed within 45 min. At increasing incubation periods, the position and width of SPR peaks remained constant, demonstrating that reaction time does not affect the size and shape of the AgNPs produced. However, when reaction time exceeded 60 min, absorption intensity decreased due to the aggregation and instability of the nanosilver. Optimum pH for the reaction conditions were determined as 9, suggesting that acidic conditions are unfavorable for AgNP synthesis. Concentration of the plant extract and AgNO_3_ solution, on the other hand, resulted in increased amounts of NP production at higher concentrations, with 6 mM AgNO_3_ and 10 mg/mL of plant extract being the suitable conditions.

Felimban et al. investigated the effect of extract concentration, temperature, pH, and light on the synthesis of AgNPs from *Olea europaea*, including both the fruit and leaves, extracts [101]. They prepared mixtures containing plant extract and 1 mM of AgNO_3_, in 1:9, 2:9 and 3:9 ratio, to investigate the effect of extract concentration on NP synthesis. UV-Vis analysis revealed 3:9 ratio samples had higher plasmon resonance bands in comparison to others, appearing at 449 and 464 nm for leaf and fruit extract, respectively. Moreover, conducting experiments at different temperatures (40, 60 and 80 °C), they have shown increased formation rate of AgNPs with increasing temperature. Smaller AgNP formation was also observed at increasing temperatures, which is attributed to the faster consumption of silver ions that minimizes the development of larger NPs. Effect of pH on the synthesis process was evaluated through three different pH values; 4 (acidic), 7 (neutral), and 9 (basic). Highest NP reaction rate was recorded at the basic environment, which might be due to the presence of a large number of functional groups (including hydroxyl groups) available for Ag binding as the authors stated. Unlike most of the studies, authors illustrated the impact of light on AgNP synthesis. UV-Vis analysis was performed to assess NP formation rates through samples grown in different settings; dark, room-light, and sunlight. Samples grown in sunlight achieved the best formation rate of AgNPs, evidenced by highest plasmon absorption bands at wavelengths of 455 and 465 nm for fruit and leaf extracts, respectively. In contrast, NP formation did not occur in dark conditions, with only negligible amounts of AgNPs produced when the fruit extract was utilized.

In a different point of view, Geetha et al. highlighted the effect of leaf samples dried using different methods, fresh, sun-dried and hot-air oven dried, on the synthesis of AgNPs from the leaf extracts of *Pimenta dioica* [102]. Preparing aqueous solutions of leaf extracts with 1 mM AgNO_3_ on four different volume ratios, 1:0.5, 1:1, 1:2 and 1:3, they were able to investigate the effect of these conditions on the NP characteristics. AgNPs prepared from the extracts of sun-dried samples in the ratio of 1:0.5 were the smallest in size, while those that were derived from fresh leaf samples in the 1:1 ratio, followed by hot-air oven dried samples in 1:1 ratio, were the largest. Also, hot-air oven dried samples in 1:1, 1:2, and 1:3 ratios yielded high numbers of AgNP production, whereas least number of AgNPs were produced from fresh leaf samples in the 1:1 ratio.

The characteristics and biological activities of green synthesized AgNPs greatly depend on various synthesis parameters including the type of the plant, AgNO_3_ concentration, and reaction conditions such as pH and temperature. Even though these factors can differ depending on specific circumstances, optimization of the green synthesis methods is crucial for the achievement of NPs with desired traits. Additionally, there is an increasing number of studies utilizing advanced statistical approaches, such as Box–Behnken and Plackett–Burman design, which can further support the optimization process by being more efficient and requiring fewer experiments in comparison to traditional methods [103,104,105].

## 4. Application of Plant-Based Synthesized AgNPs

**Figure 3:**
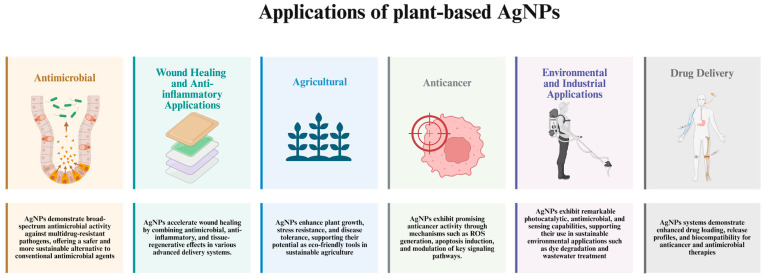
Applications of plant-based AgNPs [9,106].

AgNPs have been utilized in diverse application areas including antimicrobial, agricultural, anticancer, drug delivery, wound healing, anti-inflammatory, environmental and industrial sectors, owing to their broad-spectrum activities (Figure 3). Due to their significant antimicrobial characteristics, the latest research has been focused on the development of AgNP-included nanostructures against diverse pathogens, with a specific emphasis on their potent antibacterial activity towards both Gram-positive and Gram-negative bacterial strains. This characteristic also extends to wound healing studies where AgNPs are integrated, either solely or combined with other materials, into the wound dressings to accelerate overall healing process and prevent bacterial infections. Their ease of functionalization with various molecules led to development of drug delivery systems, as well as improving the efficiency of current treatments by facilitating a more targeted approach to anticancer drugs in addition to their own anticancer ability. In addition, their ability to regulate the expression of pro-inflammatory cytokines positions them as potential anti-inflammatory agents. Moreover, due to their anti-pathogenic mechanisms and their ability to positively affect plant growth and tolerance, AgNPs are widely applied in various agricultural applications.

### 4.1. Antimicrobial Applications

AgNPs are regarded as extraordinary antimicrobial agents with their wide spectrum of efficiency against various pathogens, including bacteria, viruses and fungi. This positions AgNPs as one of the cutting-edge topics of current nanotechnology applications, ranging from development of antimicrobial food packaging materials to formulation of antibacterial solutions [107].

However, a major drawback of these applications includes potential toxicity of AgNPs, arising from the synthesis methods. Conventional methods of AgNPs synthesis include both physical and chemical approaches, suffering from agglomeration risk of the NPs and hazardous side effects, respectively [7]. Given the increasing interest in AgNPs, specifically for their antibacterial, antifungal, antiviral, antibiofilm, and wound-healing activities, recent studies have increasingly focused on green synthesis approaches as a safer, rapid, and more sustainable alternative. Among these approaches, plant-based synthesis received particular attention due to its simplicity, cost-effectiveness, and the diverse variety of phytochemicals that can act as both reducing and stabilizing agents during AgNP formation. Also, AgNPs synthesized through plant-based routes can benefit from the intrinsic biological properties of the plant extracts, potentially improving the overall antimicrobial efficiency. These combined effects position green synthesis advantageous, as it facilitates minimization of the use of toxic chemicals while enhancing the biocompatibility and applicability of AgNPs in antimicrobial applications [108].

Hashemi et al. utilized *Berberis vulgaris* fruit extract for the synthesis of AgNPs (*BV*@AgNPs) and tested their efficiency against seven different multidrug-resistant (MDR) bacteria [109]. Spherical *BV*@AgNPs with sizes ranging from 45 to 60 nm demonstrated immense antibacterial activity against *E. coli*, *Pseudomonas aeruginosa*, *Acinetobacter baumannii*, *Enterococcus faecalis*, *S. aureus*, *Proteus mirabilis*, and *Klebsiella pneumoniae*, with MIC values of 0.4, 0.4, 0.5, 0.8, 1.56, 1.56, and 6.25 µg/mL, respectively. Cell viability assays on human breast cancer (MCF-7) and human gastric cancer (AGS) cell lines also showed *BV*@AgNPs’ anticancer activity, highlighting their comparable efficiency with chemotherapeutic agents like Doxorubicin (DOX).

Aryan et al. synthesized AgNPs from *Kalanchoe pinnata* leaf extracts and investigated their bactericidal efficiency [108]. Nearly spherical AgNPs with an average size of 38 nm exhibited strong antibacterial activity against Gram-negative *E. coli*. Following 36 h of incubation at 37 °C, results revealed AgNPs’ higher zone of inhibition (11.90 mm) in comparison to AgNO_3_ (8.80 mm) and leaf extract (7.88 mm) alone. AgNPs also had photocatalytic activity against Rhodamine B (C_28_H_31_ClN_2_O_3_), one of the most common laser dyes, under UV irradiation and in the dark. These results successfully highlighted the potential of green-synthesized AgNPs in diverse areas, serving as an eco-friendly alternative for biomedical and industrial applications.

On a deeper level, Tesfaye et al. examined the antibacterial efficiency of AgNPs green-synthesized from the leaf extract of *Vernonia amygdalina* [110]. They tested the biological activity of AgNPs synthesized at different pH values of 5 and 7, against both Gram-positive, *S. aureus and Streptococcus pyogenes*, and Gram-negative, *E. coli* and *Pseudomonas Aeruginosa* (*P. Aeruginosa*), bacteria via disk diffusion assay at different concentrations (25, 50 and 75 μg/mL). AgNPs synthesized at pH 5 led to moderate inhibition of Gram-negative bacteria with zone of inhibition values of 9, 11 and 14 mm for *E. coli* and 9, 11, and 13 mm for *P. aeruginosa* at increasing concentrations of 25, 50 and 75 μg/mL, respectively. Under the same conditions, AgNPs had increased activity against Gram-positive bacteria with zone of inhibition values of 10, 12.5, and 14 mm against *S. pyogenes* and 10, 12, and 14.5 mm against *S. aureus*. NPs synthesized at pH 7, on the other hand, were shown to be more effective in comparison to their counterparts, achieving nearly identical results with antibiotic Ciprofloxacin. At the highest tested concentration of 75 μg/mL, AgNPs had 16 and 17 mm of inhibition zones for both Gram-negative and Gram-positive bacteria, respectively.

Ghasemi et al. synthesized AgNPs from *Rubus discolor* leaf extract and highlighted their biological activities against various bacteria, including *E. coli* ATCC 8739, *P. aeruginosa* ATCC 9027 and their MDR strains [111]. AgNPs, with an average size of 37 nm and roughly spherical morphology, at a concentration of 1 mg/mL showed immense bactericidal activity with inhibition zones of 16.55 mm against *E. coli* ATCC 8739 and 18 mm against *P. aeruginosa* ATCC 9027, while aqueous *R. discolor* extract alone did not demonstrate any bactericidal activity even at a concentration of 300 mg/mL. MIC values of NPs were determined as 0.83 ±  0.2 for *P. aeruginosa* ATCC 9027 and 1.6  ±  0.11 for *E. coli* ATCC 8739. AgNPs were also found to be effective against 10 different MDR strains of *E. coli* and *P. aeruginosa*, with MIC values ranging from 1.87 to 3.75 mg/mL and 0.93 to 1.87 mg/mL, respectively, while *R. discolor* extract alone was unable to inhibit bacterial growth.

Escárcega-González et al. evaluated in vivo antimicrobial activity of AgNPs, synthesized using *Acacia rigidula* extract, on a murine infection model triggered by MDR *P. aeruginosa* strain [112]. When infected rats were treated with AgNPs, the colony forming unit (CFU) count dropped to a level similar to that of the control group, while being significantly lower than in the infected group that was not exposed to AgNPs. Further, in vivo toxicological assays on Wistar rats revealed that administering AgNPs at varying concentrations (100, 200 and 400 ppm) did not lead to adverse effects on renal and hepatic function, as the measured parameters remained statistically consistent across all treatment groups.

Subha et al. utilized *Carica papaya* leaf extract to synthesize AgNPs for the treatment of oral pathogens [103]. Spherical AgNPs, with sizes between 30 and 55 nm, were tested in terms of their antimicrobial efficiency against dental caries causing *Streptococcus* species. Specifically, solutions containing 10 µg NP concentration demonstrated excellent antibacterial activity against *Streptococcus mutans*, *Streptococcus gordonii* and *Streptococcus anginosus*, with zone of inhibition values of 10, 14, and 15 mm in diameter, respectively. These results not only highlighted the bactericidal effectiveness of green synthesized AgNPs, but also revealed their significant potential for the development of nature friendly oral health care products, including mouthwash and toothpaste, in further studies.

Ahsan et al. comprehensively evaluated the microbicidal characteristics, such as antibacterial, antifungal, and anti-inflammatory properties, of AgNPs (PrSNPs) synthesized from *Parthenium hysterophorus* leaf extract [113]. Spherical NPs with an average diameter of 20–25 nm, demonstrated antibacterial activity against *E. coli*, *S. aureus* and *B*. *subtilis*, with MIC values of 28, 31 and 43 μg/mL. Minimum bactericidal concentrations (MBC) were determined as 37 μg/mL for *S. aureus*, 38 μg/mL for *E. coli* and 45 μg/mL for *B. subtilis*. These results highlighted the enhanced bactericidal efficiency of PrSNPs compared to leaf extract alone, as well as their comparable efficiency with standard drug Ampicillin which yielded similar findings. Moreover, PrSNPs exhibited antifungal activity on *C. albicans* and *Aspergillus niger,* with MIC values of 49 μg/mL for *C. albicans,* and 57 μg/mL for *A. niger.* Despite the less efficiency of PrSNPs against fungal pathogens with respect to their bactericidal activity, authors reported that they can be employed in antifungal treatments as alternatives to common fungicidal agents such as Fluconazole. PrSNPs were also investigated in terms of their capability to treat wastewater. Results revealed a 58% reduction in *E. coli* counts, indicating the promising potential of PrSNPs for the disinfection of water. In addition, PrSNPs showed anti-inflammatory properties through the inhibition of the denaturation of Bovine Serum Albumin (BSA), even at smaller concentrations.

Hawar et al. utilized *Alhagi graecorum* leaf extracts for the green synthesis of AgNPs and further evaluated their antifungal activity [114]. Spherical shaped NPs with varying sizes, between 22 and 36 nm, successfully inhibited the proliferation of fungal pathogens belonging to *Candida* species. Results revealed AgNPs’ effectiveness against *C. albicans*, *Candida glabrata*, *Candida parapsilosis*, *Candida tropicales* and *Candida krusei*, with inhibition zones of 14 to 22 mm at a concentration of 0.01 mMol/mL. In addition, when the concentration of AgNPs were increased to 0.02 mMol/mL a corresponding increase was observed in inhibition zones, ranging from 17 to 27 mm. AgNPs also inhibited the growth of MCF-7 breast cancer cell line growth in a dose-dependent manner, with increasing concentrations from 50 to 150 150 μg/mL leading to improved results compared to the control.

In another study, Jebril et al. investigated the antifungal properties of AgNPs in both in vitro and in vivo conditions [115]. Spherical AgNPs with an average size of 23 nm showed fungicidal activity against *Verticillium dahliae*, a fungal pathogen found in eggplants. In vitro assays revealed NPs at concentrations of 20, 40, and 60 ppm hindered mycelium growth in a dose-dependent manner, with 18%, 33%, and 51% inhibition rates, respectively, compared to the control sample. For the in vivo assays, AgNPs at a concentration of 20 ppm were applied as solid drench to the soil borne *V. dahliae*, then disease severity was evaluated with respect to control samples. Following 60 days of post-inoculation, disease severity and vascular discoloration extent of AgNP treated plants were significantly reduced, respectively, by 87% and 97%, in comparison to inoculated and untreated control.

Swidan et al. conducted a comparative study on the antibiofilm activity of AgNPs synthesized using cinnamon and ginger extracts with respect to chemically synthesized NPs [116]. Spherical AgNPs with average sizes of 8.7, 41.98 and 55.7 nm for cinnamon extract, ginger extract and chemically synthesized NPs, respectively, successfully inhibited biofilm formation associated with enterococcal urinary pathogens in a dose dependent manner. Evaluating three different concentrations of AgNPs, 1/2, 1/4 and 1/8 MIC, researchers stated that biofilm formation in the presence of 1/2 MIC of cinnamon, ginger, and chemical AgNPs was 27.85%, 36.7%, and 40%. At 1/4 MIC, biofilm present in the culture increased to 49.23%, 62.6%, and 71.59%. Further, at 1/8 MIC, biofilm formed compared to the control samples was determined as 68.11%, 79.53%, and 87.48%, in the same order. To sum up, despite their comparable efficiency to chemically synthesized AgNPs at a concentration of 1/2 MIC, results revealed impressive potential of AgNPs synthesized using cinnamon extract even at lower concentrations.

Similarly, Abdelwahab et al. evaluated the antibacterial and antibiofilm activity of AgNPs synthesized using both conventional (CS-AgNPs) and green-synthesis approaches (GS-AgNPs) [117]. GS-AgNPs, those derived from curcumin, demonstrated increased antibacterial efficiency against *Acinetobacter Baumannii* (*A. baumannii*) with MIC values ranging between 7.8 and 250 μg/mL, while CS-AgNPs had MIC values falling between 500 and 1000 µg/mL. GS-AgNPs were also shown to be more efficient antibiofilm agents than the CS-AgNPs, as they successfully inhibited biofilm formation by strong, moderate, and weak biofilm-producing *A. baumannii* isolates at sub-MIC. Exposure to GS-AgNPs led to 57.43 ± 9.50%, 46.40 ± 7.24%, 39.67 ± 4.93% of strong, moderate and weak biofilm inhibitions, respectively, whereas CS-AgNPs 57.43 ± 9.50%, 46.40 ± 7.24%, 39.67 ± 4.93%, under the same tested conditions.

The antimicrobial activity of AgNPs is strongly influenced by their physicochemical properties, such as size, shape, surface charge, and surface chemistry. These properties strongly influence how particles interact with microbial cells, influencing their ability to penetrate cell membranes, disrupt cellular processes, and induce oxidative stress. Secario et al. synthesized AgNPs from green tea leaf and Cassia seed extracts, demonstrating the role of particle size in the antibacterial activity of AgNPs [118]. During the research, a significant correlation between the particle size and the antibacterial activity was highlighted. Similar research also demonstrated the variation in the size of green synthesized AgNPs depending on the plant extracts used [119]. It was found that the AgNPs synthesized from *Calendula* were nearly two times larger (35.7 ± 4.8 nm) than those synthesized from *Hyssopus* (16.8 ± 5.8 nm). During the antibacterial research, a notable difference was observed between the AgNPs, highlighting the effect of the type of plant extract and particle size on the activity of AgNPs. 

In another study, Morales-Lozoya et al. assessed the bactericidal efficiency of AgNPs having different sizes, which were green-synthesized from different parts of *Morinda citrifolia* L. [120]. Using fresh leaves, fruit pulp and dried seeds, they obtained NPs in particle sizes of 11 (AgNPs/EML), 7 (AgNPs/EMF), and 3 nm (AgNPs/EMDS), respectively. Among them, AgNPs/EMDS showed the greatest antibacterial activity, with zone of inhibition (ZOI) values of 20.45 mm for *E. coli* and 15.10 mm for *S. aureus*. In contrast, AgNPs/EMF showed ZOI values 18.13 and 14.06 mm, while AgNPs/EML had 9.81 and 10.63, against *E. coli* and *S. aureus*, respectively. This was attributed to smaller AgNPs’ higher affinity towards bacterial cell walls, facilitating easier penetration into the bacteria compared to their larger counterparts.

Shafiq et al. highlighted the effect of NP shape on the antibacterial activity of AgNPs green synthesized from *Moringa oleifera* leaf extracts [121]. By comparing spherical AgNPs with dendritic AgNPs (AgNDs), they revealed the superior antibacterial efficiency of AgNDs. Against Gram-negative *E. coli*, AgNDs had an inhibition zone of 40 ± 1.5 mm, compared to 12.6 ± 1.2 mm for spherical AgNPs. Similarly, AgNDs were also more effective against Gram-positive *S. aureus*, showing an inhibition zone of 10 ± 0.6 mm, in comparison to spherical AgNPs achieving 5 ± 0.91 mm.

These studies collectively demonstrate the importance of structure–function relationships in AgNP-based antimicrobial applications, where precise control over size and shape directly impacts their efficacy. Understanding these parameters is essential for designing targeted and optimized applications.

Apart from the antibacterial, antifungal and antibiofilm activity of green synthesized AgNPs using plant extracts, there have also been many studies underlining their virucidal activity against diverse viruses.

In a recent study, researchers investigated the antiviral activity of AgNPs, green-synthesized from green tea leaf extracts, on Newcastle disease virus (NDV) [122]. Cubic and hexagonal NPs with sizes ranging between 10 and 50 nm were tested in ovo based on their Log embryo infective dose 50 (EID_50_) to assess their effectiveness. AgNPs successfully protected embryonated chicken eggs from NDV and reduced LogEID_50_/mL values to 3.5 ± 0.4, 2.3 ± 0.6, and 2.1 ± 0.6, at their tested concentrations of 80, 160 and 320 µg/mL, respectively. The control group, on the other hand, showed a much higher LogEID_50_/mL value of 9.8. It was also demonstrated that AgNPs led to a reduction in RNA synthesis, as evidenced by RNA copy numbers per µL of 9 × 10^3^ at 40 µg/mL, 3 × 10^3^ at 80 µg/mL, 1 × 10^3^ at 160 µg/mL, and 5 × 10^2^ at 320 µg/mL.

Al-Askar et al. utilized *Punica granatum* peel extract for the synthesis of AgNPs and further tested their efficiency against Tobacco Mosaic Virus (TMV), a major pathogen affecting the growth of tomato plants worldwide [123]. AgNPs were applied on TMV-infected plants, under greenhouse conditions for 19 days, using three different timing strategies: pre-infection treatment (TB), post-infection treatment (TA) and dual-treatment (TD). Results revealed that AgNP treatment, particularly TD, reduced viral accumulation, delayed viral replication, enhanced tomato growth and upregulated the expression of pathogenesis-related genes (PR-1, PR2) as well as polyphenolic biosynthesis genes (HQT, C4H).

In another study, Dell’Annunziata et al. evaluated the antiviral potential of AgNPs synthesized from *Citrus limon* extracts, both peel (Lp-AgNPs) and juice (Lj-AgNPs), against Herpes simplex virus type 1 (HSV-1) and Severe Acute Respiratory Syndrome Coronavirus-2 (SARS-CoV-2) [124]. Spherical AgNPs with an average particle diameter of 46.1 nm (Lp-AgNPs) and 79.3 nm (Lj-AgNPs), yielded more than 50% inhibition rates at concentrations up to 7.81 µg/mL. Also, IC_50_ and IC_90_ values of 5.78–21.72 µg/mL and 6.8–24.1 µg/mL were recorded for Lj-AgNPs and Lp-AgNPs, respectively, when virulent cells and NPs were co-incubated for 1 h. Pre-treatment with the virus resulted in even more favorable outcomes, with IC_50_ and IC_90_ values of 2.29–6.13 µg/mL for Lj-AgNPs and 3.09–8.54 for Lp-AgNPs. Concerning the SARS-CoV-2, authors stated a significant inhibition of viral replication, with Lp-AgNPs and Lj-AgNPs achieving 72.5% and 71.5% inhibition rates at a concentration of 125 µg/mL in co-treatment conditions. Similar to HSV-1, increased antiviral effectiveness was observed in virus pre-treatment, with NPs reaching up to 87.5% (Lp-AgNPs) and 91% (Lj-AgNPs) inhibition rates under the same conditions.

Abo-El-Yazid et al. highlighted green-synthesized AgNPs using *Cyperus rotundus* extract as potential antiviral agents against infectious laryngotracheitis virus (ILTV) and infectious bronchitis virus (IBV) [125]. AgNPs, with sizes ranging from 11 to 19 nm and spherical morphology, significantly reduced the ILTV and IBV infected Vero cell percentage in a dose dependent manner for both pre- and post-infection treatments. Against ILTV, AgNPs demonstrated maximum antiviral activity at the highest tested dose of 50 µg/mL, in comparison to 12.5 and 25 µg/mL, supported by 27.35 ± 0.75% and 14.00 ± 4.20% of ILTV-infected Vero cell percentage following pre- and post-treatment, respectively. Similarly, against IBV, AgNPs showed comparable results with 29.10 ± 0.40% and 12.93 ± 0.80% of IBV-infected Vero cell percentage following pre- and post-treatment. However, *Cyperus rotundus* extracts alone were not successful in achieving favorable results, as they only reduced the IBV- and ILTV-infected cell percentage to 95.20 ± 0.20 and 41.07 ± 0.90%, even at a concentration of 400 µg/mL.

Current research shows that AgNPs green-synthesized via plant-mediated routes show comparable and, in many circumstances, higher antibacterial, antifungal, antibiofilm, and antiviral activity than those synthesized using conventional approaches. This promising performance can be attributed to the unique phytochemicals found in plant extracts, which not only act as reducing agents but also contribute to the stabilization and functionalization of AgNPs. With these advantages, nature-friendly and cost-effective AgNPs demonstrating enhanced antimicrobial properties can be developed for a wide array of applications, including medical device coatings, pharmaceutical formulations, industrial disinfectants and water purification systems. Still, further studies are required for the optimization of AgNPs’ synthesis parameters, long-term stability, toxicity potential and large-scale production strategies to fully maximize their potential.

### 4.2. Agricultural Applications

AgNPs exhibit significant potential for applications in agriculture, particularly in enhancing seed germination and plant growth. They can improve water uptake and seed germination while also positively influencing plant development by enhancing elemental composition, promoting root growth and shoot elongation, and inducing catalase activity [126]. As in other applications of AgNPs, green synthesis is also utilized in agriculture. Plant-based green-synthesized AgNPs are increasingly being applied in the field, including in the development of nano-pesticides and nano-fertilizers.

AgNPs are usually utilized in NP-based fertilizers as they can significantly improve the physicochemical properties of plants, while also maintaining soil fertility by preventing nutrient-loss reduction and exhibiting antimicrobial activity [127]. In the context of AgNP-based nano fertilizer applications, understanding the AgNPs’ distribution within plant tissues is essential to determine how and to what extent they influence the plant’s physicochemical properties. One study conducted a surface-enhanced Raman spectroscopy-based monitoring to reveal where these particles tend to accumulate in maize seedlings [128]. The results suggested that administered AgNPs are accumulated in the epidermis and cortex of the root and phloem parts of the shoot, demonstrating a systemic transport of the particles within the plants. Depending on the uptake route of AgNPs (such as foliar or root), the mechanism and internalization of AgNPs may differ [129]. This factor can also be influenced by the physicochemical property of the particle, such as the larger size of AgNPs can lead into formation of additional pores in the plasma membrane, while small-sized particles can directly pass through the cell membrane [130]. Depending on the accumulation pattern, it is possible that AgNPs can influence key physicochemical processes related to nutrient transport and metabolic activity, supporting their potential application for enhancing plant growth and crop yield.

Ansari et al. increased the growth and yield of tomato plants against early blight disease, using synthesizing AgNPs from leaf extract of the *Azadirachta indica* (*A. indica*) [131]. The AgNPs treatment increased several features of tomato plants: increased plant height by 30%, fresh weight by 45%, number of leaves and dry weight. Moreover, it was recorded that AgNPs reduced disease severity index by 73% and disease incidence by 69%, while increasing the secondary metabolite accumulation and improving stress tolerance of the plants. In a follow-up study by the same research group, AgNPs synthesized via the same plant-based method were also shown to enhance shoot length, plant biomass, and the production of chlorophyll, proteins, and flavonoids [132]. Sabir et al. synthesized AgNPs using *Moringa oleifera* leaf extract, testing its potential on wheat plants against stripe rust disease [133]. Although AgNPs did not completely inhibit disease progression, they significantly reduced the disease index by 80%. In addition, several physicochemical properties of the infected plants were positively affected, including non-enzymatic attributes, protein, sugar, and flavonoid content, as well as enhanced antioxidant response and resistance capacity. Haq et al. enhanced physicochemical properties and yield of sunflower plants using green synthesized AgNPs from *Mentha arvensis* plants [134]. The AgNPs were applied with foliar application at a concentration of 25 mg/L, leading to significant improvements in the following properties: a 70.4% increase in the number of leaves, a 35.2% increase in shoot length, increases of 57.3% and 30.3% in the fresh weights of shoot and root, and increases of 62.08% and 22.8% in the dry weights of the shoot and root, respectively. Moreover, the same application also increased the following plant components: carotenoid content by approximately 271.8%, chlorophyll a content by approximately 196.1%, chlorophyll b content by approximately 182.3%, total soluble protein by 26.8%, increase in soluble sugar content by 42.3%, proline content by 76.1%, phenol content by 71.5%, and the activity of several antioxidant enzymes ranging between 19.08% and 301.6%. Finally, the head diameter of sunflowers increased by 62.73%, seed yield per plant increased by approximately 58.2%, oil content increased by 21.5%, and seed protein content increased by approximately 33.02%.

Antimicrobial activity is one of the key features of AgNPs that are strongly utilized in agricultural applications. The potent antimicrobial activity of AgNPs allows their use as effective agents against a broad spectrum of plant pathogens. Even though not specifically clarified, the AgNPs might be able to interact with both plant pathogens and plant components, resulting in favorable conditions for the plants during the infection. The AgNPs can affect plant immunity through influencing stress-related signaling pathways, inducing expression of defense-related genes and regulating defense mechanism-associated compounds [135]. From another perspective, the molecular mechanism behind the interaction of AgNPs and phytopathogens is not fully understood. Still, similar to the generally proposed antimicrobial mechanisms of AgNPs, their activity against phytopathogens potentially involve multiple, related pathways such as ROS generation, disruption of membrane integrity, promotion of intracellular penetration, and negatively affecting gene expressions [136,137]. It should also be noted that these potential pathways might act simultaneously against various phytopathogens, highlighting the need for comprehensive mechanistic investigations to unravel their synergistic effects and better understand the broad-spectrum antimicrobial potential of AgNPs.

Rana et al. synthesized AgNPs with *A. indica* and *Mangifera indica (M. indica)* tree leaf extracts to evaluate their antimicrobial activity against various plant pathogens [138]. Both types of AgNPs exhibited notable antifungal and antibacterial effects, along with concentration-dependent antioxidant activity. The choice of tree leaf extract significantly influenced the physicochemical properties and bioactivity of the synthesized particles. AgNPs derived from *A. indica* had lower particle size, a slightly lower zeta potential, and an inhibition zone nearly 50% larger compared to those synthesized from *M. indica*. El-Ashmouny et al. synthesized AgNP using *Artemisia herba-alba* plant extract to investigate its larvicidal activity against the cotton pest *Spodoptera littoralis* larvae [139]. The AgNPs exhibited larvicidal effects, as the mortality rate of the larvae increased proportionally with higher NP concentrations. Histological and morphological analyses revealed the potential mechanism of action, highlighting structural damage such as the destruction of the cuticle layer and musculature of the gut. Elkobrosy et al. demonstrated the nematocidal and bactericidal activity of AgNPs synthesized from aqueous extract of *Ficus sycomorus* leaves [140]. Against plant pathogenic bacteria and root-knot nematodes, green-synthesized AgNPs demonstrated high mortality and inhibition ratio with an increasing concentration. Tian et al. used three different plant extract sources, *Arctium lappa* fruit, *Solanum melongena* leaves, and *Taraxacum mongolicum* leaves, in AgNPs synthesis to determine their antipathogenic potential against rice bacterial leaf blight pathogen *Xanthomonas oryzae* pv. *oryzae* [141]. The synthesized AgNPs expressed a slight difference in their size among them, as particles synthesized *Taraxacum mongolicum* leaves demonstrated approximately 40.08 nm, while the particle sizes are halved for the other two particles, 20.18 nm and 21.00 nm, respectively. Considering the other slight differences in their physicochemical properties, AgNPs with the larger particle size demonstrated the lowest inhibition zone, decrease in bacterial growth, prevention of biofilm formation and highest bacterial number after the treatment (in terms of optical density value). Still, all three types of green synthesized AgNPs exhibited notable antipathogenic activity, further supporting their potential application in controlling plant diseases.

Green-synthesized AgNPs can be applied to increase the shelf life of agricultural products by reducing microbial spoilage and controlling foodborne pathogens. Considering their broad-spectrum of antimicrobial activity, AgNPs are strong alternatives as antimicrobial coatings for food packages. While AgNPs can enhance the important features of food packages, such as mechanical and barrier properties, the green synthesized methods can neglect the toxicity concerns of the particles.

AgNPs synthesized from *Eucalyptus* leaf extract significantly extended the shelf life of bananas while enhancing their morphological and physiological properties [142]. Treatment of 0.01% AgNPs significantly extended the shelf life of bananas to 32 days, effectively slowing the ripening process and reducing weight loss. Additionally, AgNP treatment contributed to preserving the fruit’s color, enhancing firmness, and maintaining pH levels. In addition, a higher plant extract concentration was found to correlate with increased absorbance peak intensity and larger particle size during the synthesis. Another study synthesized AgNPs from puerh tea leaves, demonstrating their antibacterial activity against various strains of Gram-negative foodborne pathogens [143]. While AgNP treatment demonstrated significant MBC and MIC values, ranging from 3.9 to 7.8 μg/mL showing the effective bactericidal activity of the particles, the disk diffusion test also revealed the inhibition of pathogen growth. Gopalakrishnan et al., synthesized AgNPs using pomegranate and citrus fruit peel, modifying cellulose-based wrapping materials for extending the shelf-life of packaged bread [144]. Compared to the control group, whereas only the peel extracts are used, synthesized AgNPs demonstrated antioxidant activity, increased weight and film density, notable decrease in oxygen and water vapor permeability and higher hydrophobicity. Moreover, the AgNPs addition induced delayed growth of microbial count in the bread packages up to 7 days, highlighting their activity as antimicrobial coating. Similarly, AgNPs synthesized from *Azadirachta indica* leaf extracts were added into nanocomposite film and successfully acted as an antimicrobial packaging material [145]. The synthesized particles expressed potent antibacterial activity against several bacteria strains by forming zones of inhibition between 26 and 33 mm. The designed nano composite films were tested on eggplants, demonstrating protection of the plant from rotting up to 15 days compared to AgNP-uncoated films. Several physicochemical properties of the eggplants, such as texture, shading, and appearance, were also preserved during the experimentation.

The green synthesized AgNPs represent a promising and multifunctional agent in agricultural applications. Their potential extends across food packaging development to increase shelf life of products by preventing microbial spoilage and inhibiting growth of foodborne pathogens, potential agent in plant disease management, through their potent antimicrobial activity against a wide range of phytopathogens and enhancing physicochemical properties and crop yields with AgNPs-based nanofertilizers. The use of plant-derived synthesis not only contributes to environmental safety but also improves economic feasibility. Together, these benefits highlight the potential of plant-based AgNPs to advance agricultural productivity, ensure food safety, and promote eco-friendly farming practices.

### 4.3. Anticancer Applications

One of the current focuses of current nanotechnology research is to develop anticancer drugs from metallic NPs, especially those derived from gold, silver and platinum. Among these, AgNPs take a step forward in comparison to their counterparts due to their unique properties, including ease of functionalization, high stability, inherent antimicrobial characteristics as well as cost-effectivity [146]. However, despite these rapid advances in nanotechnology, only a very small number of nanomaterials are considered suitable in biomedical applications as anticancer drugs due to potential toxicity and safety concerns. For this reason, green synthesis, especially plant-based methods, emerges as a non-toxic, biocompatible and sustainable alternative to overcome these limitations. Apart from avoiding the use of hazardous chemicals and reducing the overall toxicity, plant-mediated green synthesis also enhances the therapeutic value of AgNPs due to the presence of a wide range of phytochemicals that can act as natural reducing and stabilizing agents. This contributes to AgNPs selectivity and cytotoxicity against cancer cells, offering new prospects for safer and more effective anticancer nanotherapeutics [92].

ROS production is thought to be the primary mechanism behind AgNPs’ anticancer activity. AgNPs may inhibit the growth and viability of cancer cells by damaging their ultrastructure, which induces the formation of ROS and eventually leads to DNA damage [147]. Also, researchers stated that AgNPs have the capability to activate caspase-3 and caspase-9, two key executioner enzymes in the apoptotic pathway that induce programmed cell death. Accordingly, it was revealed that AgNPs green-synthesized from *Lonicera hypoglauca* flowers increased the expression of both caspases on MCF-7 cells compared to the control samples, eventually leading to induction of apoptosis. The study also revealed the effect of AgNPs on JAK/STAT signaling, which has been known to be involved in the initiation and progression of cancer by up-regulating anti-apoptotic genes. Results revealed that samples treated with NPs successfully inhibited mRNA expression of JAK, STAT-1 and STAT-3 compared with untreated control samples [148].

In another study, authors pointed out a different mechanism where AgNPs synthesized from the whole plant of *Bergenia ligulata* induced expression of tumor suppressor protein p53 in a dose-dependent manner. Their results showed a gradual decrease in Bcl-2 levels, along with a simultaneous increase in Bax expression in breast cancer cells treated with green-synthesized AgNPs via the plant-mediated route. These findings align with the well-known role of p53, that of promoting apoptosis by upregulating the expression of the pro-apoptotic gene Bax and downregulating anti-apoptotic genes such as Bcl-2 [149]. Xu et al., on the other hand, highlighted that AgNPs produced from aqueous extracts of *Ginkgo biloba* showed anticancer effects through a mitochondrial pathway by inducing the release of cytochrome c, along with leading to an increase in ROS levels and p53 induction [150]. They have shown that expression of cytochrome c in the cytosol greatly increased through treatment with AgNPs on both HeLa and SiHa cells in comparison to control samples.

Hashemi et al. synthesized AgNPs from *Teucrium polium* leaf extracts and further evaluated their antitumor properties on human gastric cancer cell line (MNK45) [151]. Spherical and nearly spherical AgNPs with sizes ranging from 70 to 100 nm, demonstrated dose-dependent anti-proliferative activity on MNK45 cells at different concentrations of 12.5, 25, 75 and 130 μg/mL. When the concentration of *T. polium*-AgNPs increased up to 130 μg/mL, cancerous cells had a cell viability of only 26.1%. Moreover, researchers determined the IC_50_ value of NPs on the MNK45 cell as 68.2 μg/mL, following 48 h of exposure at 37 °C [151].

Alharbi et al. utilized *Azadirachta indica* fruit extracts for the production of AgNPs, then investigated their in vitro anticancer efficiency on human lung adenocarcinoma cell line (A549) [152]. Comparing three different types of AgNPs; *A. indica*-AgNPs, AgNPs combined with fruit extract (AgNPs-E) and AgNPs with chemotherapeutic drug cisplatin (AgNPs-cis) at varying concentrations, 12.5, 25, 50, 100, 150 and 200 μg/mL, researchers assessed the anticancer efficiency following 48 h of incubation. Results revealed all three types of AgNPs’ efficiency in inhibiting the viability of cancerous cells, in comparison to extract or cisplatin alone, which is thought to be occurring from the NPs’ ability to stimulate apoptosis. It was also highlighted that AgNPs had greater anticancer activity against A549 cells compared to AgNPs-E, with an IC_50_ value of 64.46 μg/mL, likely due to their smaller particle size which facilitates more efficient penetration through the cell membrane. Further, microscopy analysis and 4′,6-diamidino-2-phenylindole (DAPI) staining showed exposure to AgNPs led to disruption in the morphology of cancerous cells along with nuclear shrinkage, fragmented DNA and chromatin condensation, while untreated control samples exhibiting typical pebble-shape with normal nuclei and undamaged cell membranes.

Venkatadri et al. synthesized AgNPs from the aqueous extracts of *Curcuma longa* and *Zingiber officinale* rhizomes [153]. NPs within a size range of 20 to 61 nm and a spherical shape were tested for their anticancer efficiency on human colon carcinoma (HT-29) cells through MTT assay. Results revealed that AgNPs, ranging from 25 to 500 μg/mL, were cytotoxic to HT-29 cells with increasing concentrations leading to more potent decrease in cell viability, following 24 h of exposure at 37 °C. In addition, IC_50_ value of green-synthesized AgNPs determined as 150.8 µg/mL, further supporting their effectiveness [154]. In another study, Al Baloushi et al. produced AgNPs from aqueous leaf extracts of *Moringa peregrina.* NPs with varying sizes, between 30 and 35 nm, exhibited anticancer effects on breast cancer (MCF-7) and colorectal cancer (Caco-2) cell lines, with IC_50_ values of 26.93 and 41.59 µg/mL, respectively.

Antony et al. investigated in vivo antitumor efficiency of AgNPs synthesized using *Ficus religiosa*, which have been shown to possess anticancer activity against breast cancer cells, for the treatment of Dalton’s ascites lymphoma (DAL) [155]. Spherical NPs within the size range of 5 to 35 nm were found to be efficient in controlling tumor growth at a concentration of 50 µg/mL, shown by reduced tumor volumes, decreasing ascites fluid accumulation and increased life span of DAL induced mice treated with AgNPs. Also, AgNPs exhibited apoptosis inducing and anti-angiogenic properties without affecting the normal functions of the kidney.

Amini et al. utilized *Teucrium polium*, a wild-growing herb, rich in terpenoids, iridoids, and flavonoids, for the synthesis of AgNPs [156]. Circular NPs with an average size of 14.3 ± 9.7 nm were exposed to the B cell precursor leukemia cell line (NALM-6) at varying concentrations (1, 2.5, 5, 10, 25, 50, 100, and 200 µg/mL) following 24 and 48 h of treatment. Results revealed that increasing concentrations and longer incubation times led to enhanced outcomes, as indicated by decreased viability of NALM-6 cancer cells, with 200 µg/mL of AgNPs showing the most significant effect after 48 h of exposure. Authors also compared the anticancer efficacy of the NPs with that of the plant extract alone. After 24 h of treatment, the plant extract showed comparable effectiveness to AgNPs. However, during the 48 h of treatment, the extract was unable to match the cytotoxic effects achieved by the AgNPs even at their highest concentration of 500 µg/mL.

One major field where AgNPs show great potential is theranostic applications. Considering their notable optical properties and high cytotoxic activity against various cancer cell lines, AgNPs can potentially be utilized for both diagnostic and therapeutic purposes, particularly in anticancer studies [157]. Several research and review articles have highlighted the potential of AgNPs in theranostics [4,158,159,160]; however, a solid experimental foundation for their dual functionality has not yet been fully established. This gap highlights the need for future research focused on integrating imaging modalities or biosensing capabilities with AgNP-based platforms to fully realize their theranostic potential.

It is also important to mention that the anticancer efficiency of AgNPs is highly dependent on the type of cancer, as variations in cellular architecture, signaling pathways and redox environment influence NP uptake and cytotoxic mechanisms. For instance, while lung cancer cells often exhibit altered redox balance, mitochondrial dysfunction and upregulated survival pathways like EGFR/p38 and PI3K/AKT, AgNPs have been shown to induce apoptosis primarily via ROS generation, mitochondrial membrane disruption and activation of caspase cascades. In contrast, other cancer types like breast or cervical cancer, may respond differently due to their distinct expression profiles of apoptotic regulators, antioxidant defenses or membrane transport proteins. These cancer-specific features ultimately determine the extent and mechanism of AgNP-induced cytotoxicity, highlighting the need for individualized evaluation of AgNPs on different cancer models [161]. In line with that, Venmani et al. assessed the anticancer activity of AgNPs, green synthesized from leaf extracts of *Cymodocea serrulata*, against four different cancer cell lines: human breast cancer (MCF-7 and MDA-MBA-231), human lung cancer (A549) and hepatic cancer (HepG2) [162]. Spherical AgNPs with an average size of 30.5 ± 2.5 nm exhibited cancer-specific activity, showing highest potency against MCF-7 cell line with an IC_50_ value of 57.3 ± 2.5 μg/mL, compared to the rest of cell lines with IC_50_ values of 82.5 ± 3.7 (MDA-MB-231), 87.6 ± 4.1 (HepG2) and 93.4 ± 4.5 (A549). Further, researchers revealed that AgNPs led to distinct morphological changes in these cell lines following incubation at their corresponding IC_50_ concentrations, which is potentially due to the enhanced cellular uptake through endocytosis and retention of AgNPs.

Similarly, Meti et al. evaluated the anticancer potential of AgNPs green synthesized from seed extracts of *Acacia sinuata*, against human colon adenocarcinoma cells (Caco-2) and human osteosarcoma cells (MG-63) [163]. Spherical AgNPs, having an average diameter of 15.54 ± 5.36 nm, showed strong effectiveness against Caco-2 cell lines, with an IC_50_ value of 1.03 ± 0.13 μg/mL. In contrast, their activity against MG-63 was modetarte, indicated by an IC_50_ of 21.03 ± 0.24 μg/mL. At a concentration of 100 μg AgNPs reduced cell viability to 37.51% and 56.94% in Caco-2 and MG-63 cells, respectively, further highlighting their selective cytotoxicity towards various cancer cell lines.

Together, these studies demonstrate that green-synthesized AgNPs through plant-mediated approaches exert their anticancer effects through multiple molecular pathways, including ROS generation, apoptotic enzyme activation, modulation of key signaling cascades and regulation of tumor suppressor proteins.

### 4.4. Wound Healing and Anti-İnflammatory Applications

The AgNPs have gained significant attention in the field of regenerative medicine, regarding their significant antimicrobial activity and potential in anti-inflammatory applications. AgNPs not only offer a potent antimicrobial activity that inhibits any pathogenic activity in the wound area, but also possess the ability to module cellular and molecular pathways that influence tissue repair and modulate anti-inflammatory mechanisms [9]. One major drawback for the wide-ranging application of AgNPs in the area is their cytotoxicity potential. Unlike conventional chemical methods, using green synthesis methods reduces the major cytotoxicity problem and gives the necessary biocompatibility for wound healing applications [164]. It has been suggested that AgNPs can increase wound closure speed, promote re-epithelialization and module inflammatory responses through modulating cytokine expression and controlling oxidative stress levels [165]. As a result, green-synthesized AgNPs have been incorporated into wound healing applications in various forms, including hydrogels, films, coatings, and nanofibers [166]. Owing to their combined antimicrobial, anti-inflammatory, and regenerative properties, plant-derived AgNPs are emerging as promising multifunctional agents in advanced wound care strategies.

Likkem et al. highlighted the effectiveness of green synthesized AgNPs from extracts of *Catharanthus roseus* and *Azadirachta indica* in wound healing [167]. Confirming the antibacterial activity of AgNPs on both Gram-positive and Gram-negative bacteria, with the highest activity observed against MDR *K. Pneumoniae* exhibiting zone of inhibition values of 16 mm for *C. roseus*-AgNPs and 19 mm for *A. indica*-AgNPs, authors further conducted in vivo studies on BALB/c mice. Results revealed enhanced wound constriction efficacy compared to control groups, along with the prevention of microbial growth, hemorrhage and formation of pus throughout the 11 days of treatment. During the process, AgNP treated mice had better wound healing capacity and decreased wound size, with *C. Roseus*- and *A. indica*-AgNPs reaching up to 94% ± 1% and 87% ± 1% wound healing, respectively, while the control group had 74% ± 1%. Assad et al. synthesized AgNPs from the extracts of the *Cotoneaster nummularia* leaves [168]. NPs with an average diameter of 122.8 ± 1.1 nm and a cubical morphology demonstrated antimicrobial activity against pathogenic bacteria (*E. coli*, *S. epidermidis* and *K. pneumoniae*) and fungi (*Aspergillus niger* and *Aspergillus flavus*), in a comparable level with standards. Further in vivo studies revealed the promising wound healing potential of AgNPs. Rabbits exposed to NPs had 2.41%, 27.73%, 73.21%, 96.22% and 100% wound closure, on 1st, 2nd, 6th, 9th, and 12th days of the treatment, respectively. These results were comparable to the standard bandages which achieved 3.19%, 30.50%, 75.56%, 97.89% and 100% closure rates under the same conditions.

Apart from the direct application of AgNPs to the wound site, recent research highlighted novel strategies such as the development of green synthesized AgNPs-incorporated hydrogels, biocomposite films or AgNP-coated biomaterials to promote wound healing.

For instance, Barathi et al. demonstrated the wound healing activity of chitosan hydrogels loaded with AgNPs synthesized from *Saussurea lappa* (Sl) aqueous root extract [169]. In their study, Wistar rats treated with SI-AgNP hydrogel showed immense wound healing capability, along with lower bacterial counts and increased production of connective tissues, at a concentration of 0.1 mg of Sl AgNPs/g hydrogel during the application. *P. aeruginosa* counts on NP treated rats were determined as 2 × 10^5^ CFU/10 µL at day 5. By day 10, this count had dropped to 3 × 10^3^ CFU/10 µL, eventually reaching zero by day 15. The wound contraction study of SI-AgNPs hydrogel further supported these findings, highlighting reductions in wound contraction means of 518 ± 13.3 mm^2^ at the start, 321 ± 11.78 mm^2^ on day 5, 94 ± 1.11 mm^2^ on day 10, and 16 ± 0.47 mm^2^ on day 15, as revealed by scanning electron microscopy analysis. From another perspective, Maghimaa et al. developed AgNP coated cotton fabrics for antimicrobial applications and wound healing activity [170]. AgNPs synthesized from aqueous extracts of *Curcuma longa* leaves, with sizes ranging from 15 to 40 nm and spherical morphology, showed strong antibacterial and antifungal activity against skin infection causing pathogens. Specifically, NPs had MIC values of 5 μL for *S. pyogenes*, 2.5 μL for *S. aureus* and *E. coli*, 2.25 μL for *P. aeruginosa* and 1.25 μL for *C. albicans.* Further, AgNPs loaded cotton fabrics were examined through an in vitro wound scratch assay on fibroblast (L929) cells for their wound healing capability. Wounded cells supplemented with 20 μg of AgNPs had enhanced migration towards the wounded area, in comparison to both untreated control and the 15 μg AgNP-containing group after 24 h of incubation at 37 °C. These results not only demonstrated the promising potential of AgNPs integrated into cotton fabrics for biomedical applications but also offered a novel approach for the prevention of microbial infections.

Gupta et al., on the other hand, developed nanofilms containing AgNPs synthesized from the aqueous extracts of *Ocimum sanctum* for wound healing applications. Spherical NPs with an average size of 28.95 ± 7.74 nm were utilized at different concentrations; 0% *w*/*v* (CG), 1% *w*/*v* (CG_1_), and 2% *w*/*v* (CG_2_) in the production of chitosan-gelatin nanocomposite films. Through antibacterial analysis, it was revealed that increasing concentrations of AgNPs led to improvements in bactericidal efficiency, with CG_2_ exhibiting a zone of inhibition of 9.68 ± 0.77 mm, compared to 7.46 ± 0.50 mm for CG_1_ and 7.00 ± 0.29 mm for CG against *E. coli.* Evaluating the wound healing activity of nanofilms via in vitro scratch assay on L929 cell line over 12 h, the authors found the highest wound closure rate of 45% with CG_1_, showing higher efficiency than CG which had 40% closure. In contrast, CG_2_ was found to be less efficient than both CG_1_ and CG_2_, likely due to the slight toxicity arising from increased AgNP concentrations as the authors stated [171].

The anti-inflammatory activity of AgNPs is one of the primary attributes that strengthens their effectiveness in wound healing applications. In the context of tissue repair, the regulation of inflammatory responses is critically important, as prolonged or excessive inflammation can not only impair the healing process but also lead to chronic wounds and secondary tissue damage [166]. Green-synthesized AgNPs can reduce the expression of pro-inflammatory cytokines, such as TNF-α, IL-1β, and IL-6 and influence gene expression like COX-2 [172]. This dual modulation enables wide-ranging utilization of the anti-inflammatory properties of AgNPs, not only in tissue regeneration studies but also as potential agents in the treatment of various inflammation-associated disorders.

Bold et al., conducted an in vivo study to demonstrate the anti-inflammatory and wound healing capacity of AgNPs synthesized from *Rhodiola rosea* [173]. The AgNP treatment in post-burn injury of the murine model demonstrated that the particles significantly reduced the pro-inflammatory cytokine levels (IL-6, IL-1, and TNF-α) by more than 50%, while increasing IL-10 to 1.5-fold. With the enhancement of the anti-inflammatory activity, the AgNPs demonstrated promising results on the mice wounds, reducing the burn wound surface area from 61 ± 9 mm^2^ to 5 ± 4 mm^2^. Tyavambiza et al., demonstrated the anti-inflammatory property of AgNPs synthesized with *Cotyledon orbiculata* aqueous extract [174]. The results demonstrated that AgNPs treatment reduced cytokine release (TNF-α, IL-1β and IL-6) up to 10.5-fold in THP-1 cells. Another study highlighted the decreased expression of iNOS and COX-2 by green-synthesized AgNPs from black roasted gram (*Cicer arietinum*) [175]. The dose-dependent anti-inflammatory activity of AgNPs significantly reduced the protein expression of iNOS and COX-2 by approximately 5-fold and 2.5-fold in LPS-stimulated RAW 264.7 macrophages, respectively. Similarly, AgNPs synthesized with *Laurus nobilis* leaves demonstrated anti-inflammatory activity by reducing induction of inflammation in human leukemic monocytes [176]. The study compared chemically synthesized AgNPs with green-synthesized counterparts in terms of their effects on pro-inflammatory markers, including IL-6, IL-8, COX-2, and TNF-α expression. The findings revealed that conventional AgNPs triggered significantly higher inflammatory responses, whereas green-synthesized AgNPs maintained cytokine levels within physiologically acceptable ranges, indicating a more biocompatible and less immunogenic profile. Such a comparison clearly highlights the advantages of green synthesis in enhancing the biocompatibility of AgNPs, particularly for inflammation-sensitive applications.

### 4.5. Environmental and Industrial Applications

The multifunctional characteristics of AgNPs allow for their diverse utilization in environmental applications. Primarily due to their antibacterial activity, AgNPs exhibit significant potential in water purification, wastewater treatment, dye removal, air disinfection, and related areas [177]. In particular, the green synthesis of AgNPs, especially plant-based approaches, represents a promising strategy aligned with the principles of green chemistry. Beyond antimicrobial effects, their photocatalytic activity further strengthens their potential in these applications.

The optical properties of AgNPs, particularly their SPR, play a critical role in photocatalytic applications. Under light irradiation, the excitation of surface electrons can generate reactive species capable of degrading dye molecules, making plant-based AgNPs highly effective in these applications [178]. In addition to light-driven photocatalysis, AgNPs can also act as efficient catalysts through electron transfer processes, thereby enhancing the degradation of dye molecules [179].

David and Moldovan demonstrated the catalytic potential of AgNPs synthesized from *Viburnum opulus* fruit extract for the degradation of harmful organic dyes, including tartrazine, carmoisine, and brilliant blue FCF mediated by NaBH_4_. The spherical AgNPs, averaging 16 nm in diameter, exhibited dose-dependent catalytic activity. Increasing AgNP concentrations (20, 40, and 60 µg/mL) corresponded to higher dye degradation rates. Incorporation of AgNPs at a concentration of 60 µg/mL effectively degraded carmoıisine, achieving a reaction rate three to five times faster than NaBH_4_ alone. In the case of tartrazine, only 28% degradation occurred after 1 h without AgNPs. However, the inclusion of AgNPs to the reaction medium boosted the degradation rate approximately four-fold. AgNPs had the highest catalytic efficiency observed against brilliant blue FCF, with accelerating the reaction rate by a remarkable 27-fold while NaBH_4_ alone degraded only 38% of the dye after 1 h [180].

Kadam et al. synthesized AgNPs from cauliflower (*Brassica oleracea* var. *botrytis*) waste extract and demonstrated their potential in Hg^2+^ biosensing along with significant photocatalytic activity [181]. The photocatalytic activity of AgNPs was tested with sunlight exposure against methylene blue, which the degradation rate was found to be 97.57% at 150 min. The biosensing activity of AgNPs against Hg^2+^ was tested along with several other metal cations, such as Co^2+^, Pb^2+^, Mn^2+^, Zn^+2^ and Mg^+2^. Upon testing, it was revealed that interaction of AgNPs with Hg^+2^ ions induced a significant color change from brown to pale yellow, whereas other metal cations did not cause such a change. The authors suggested that this observable color change might result from a redox reaction, as a notable reduction in absorbance intensity was observed upon interaction. Dua et al. characterized AgNPs synthesized from *Eupatorium adenophorum* leaf extract, an evaluation of their photocatalytic activity [182]. The degradation of Rhodamine B was observed under sunlight irradiation, with the AgNPs showing an absorption peak at 554 nm. The treatment resulted in a photodegradation efficiency of 78.69%, followed by notable decolorization after 90 min.

Similarly, Aryan et al. synthesized AgNPs from *Kalanchoe pinnata* leaf extracts and demonstrated their photocatalytic activity against Rhodamine B under UV irradiation [108]. The photocatalytic degradation efficiency of AgNPs was evaluated under both dark conditions and UV irradiation. Under UV light, rapid and efficient degradation was observed, achieving 83% degradation within 45 min and resulting in a color change in the dye solution from pink to nearly colorless. In contrast, the degradation process under dark conditions was notably slower, with 87% degradation achieved after 150 min. Additionally, a shift in the SPR band during UV irradiation was observed, highlighting the role of the optical properties of AgNPs during the process. Another study demonstrated the photocatalytic activity of AgNPs synthesized from aqueous extract of *Pelargonium hortorum* flower and *Allium fistulosum* against azo dyes [183]. Both types of AgNPs demonstrated significant dye degradation activity against blue and green dyes, achieving degradation efficiencies of up to nearly 100%. The degradation process was notably faster for AgNPs synthesized from *Allium fistulosum*, leading to almost complete degradation within 5 min. In contrast, AgNPs synthesized from *Pelargonium hortorum* flower reached 85% efficiency within 15 min and achieved a maximum of 95% after 240 min.

AgNPs have gained significant attention as effective agents for wastewater treatment. Their unique characteristic properties, especially their unique SPR, potent antibacterial activity and strong catalytic efficiency, making them highly suitable for disinfection methods [9]. Specifically, plant-based synthesis of AgNPs can offer an alternative to conventioınal treatment methods within these areas.

Muñoz-Carrillo et al. produced AgNPs from *Opuntia ficus indica* fruit peel extracts and further evaluated their ability to treat wastewater as an alternative to commonly used process chlorination, which is associated with the production of toxic byproducts. Spherical AgNPs spherical, with an average particle size distribution of 64.28 ± 11.82 nm, successfully inhibited the growth of coliform bacteria, including *E. coli*, *Enterobacter aerogenes*, *Citrobacter freudi* and aerobic mesophilic microorganism. After 30 min of exposure to 0.5 mg/mL AgNPs, the concentration of both fecal and total coliform bacteria was determined as 0.03 MPN/100 mL, indicating their absence. However, samples subjected to chlorination had a total coliform count of 91 MPN/100 mL, reflecting the presence of microorganisms. Authors stated that chlorination was able to inhibit proliferation of approximately 90% aerobic mesophyll bacteria, while AgNPs achieved up to 99% inhibition. These highlighted the promising potential of AgNPs as effective and safer alternatives to traditional chlorination in wastewater treatment [184].

Similarly, Raota et al. utilized *Vitis labrusca* for the production of AgNPs and investigated their ability to inhibit proliferation of Gram-negative bacteria present in wastewater [185]. Researchers immobilized AgNPs into a polymer matrix of chitosan with the aim of facilitating easy removal and recovery of NPs following the treatment. Further, 250 mL of wastewater contaminated by approximately 40 CFU/mL *E. coli* exposed to chitosan-AgNPs. After 1 h of incubation, the number of CFU/mL started to reduce, achieving 47% reduction in initial bacterial count.

Shittu et al. synthesized AgNPs from *Piliostigma thonningii* aqueous leaf extract for wastewater purification [186]. Spherical NPs with sizes varying from 50 to 114 nm exhibited pH- and contact time-dependent removal of metal ions. At the highest tested pH of 9, AgNPs successfully removed magnesium, copper and iron ions with a removal percentage of 93.6%, 89% and 95%, respectively, in comparison to pH 3 and 7. Similarly, increasing the contact time to 60 min enhanced metal ion removal, reaching 71.8% for magnesium, 82.1% for copper, and 96.9% for iron. AgNPs were also effective in removing heavy metal ions, such as lead. Highest removal efficiency was observed at pH 3, achieving a removal rate of 96.8%. Increased contact time further improved removal of lead ions, achieving up to 97.89%, following 1 h of incubation in comparison to shorter durations of 20 and 40 min.

Aboelghait et al. utilized *Murcott Mandarin* peel extracts to synthesize AgNPs for the removal of lead ions from industrial wastewater. Following batch experiments, AgNPs had exceptional removal capability of lead ions at a rate of 42.7 mg/g (under optimal conditions of pH 5.5 and 1 h of stirring), outperforming the *Murcott mandarin* solid residue which achieved only 6.6 mg/g under the same optimal conditions [187].

Another promising area for the application of green-synthesized AgNPs is in optical and sensing technologies. Owing to their unique SPR properties and the enhanced biocompatibility through green synthesis, AgNPs can be extensively utilized in various optic-based applications, including biosensing, bioimaging, and photocatalysis [188,189]. Recent studies have demonstrated that plant-derived AgNPs can significantly enhance sensor sensitivity and detection capability, thereby contributing to the advancement of sustainable optical devices. The optical properties of AgNPs can be utilized in biosensor development, as their surface interactions with biomolecules induce changes in optical signals, enabling sensitive and selective detection [190].

One study utilized carbon paste electrodes modified with green-synthesized AgNPs synthesized with black tea leaf extracts to develop an amperometric biosensor for glucose detection [191]. The designed biosensor exhibited promising analytical performance, with a notably low limit of detection of 0.016 μM and response time of 200 s. Glucose determination was performed in fruit juice samples, and the sensor’s selectivity was evaluated using ascorbic acid as a potential interferer. Although ascorbic acid caused an interference of only 1.09%, the biosensor maintained a detection accuracy of 99.98%. A similar study synthesized AgNPs with rhizome essential oil (*Curcum Longa*) to develop enzyme-functionalized optical biosensor for ascorbic acid detection [192]. The designed biosensor demonstrated a notable detection range for ascorbic acid, ranging from 110 μM to 400 μM. Furthermore, selectivity testing revealed high specificity of the biosensor toward ascorbic acid compared to glucose, urea, vitamin B, and dopamine. Rashidi et al. synthesized AgNPs from *Smyrnium cordifolium* extract and demonstrated their application for colorimetric detection of ammonia [193]. The interaction between AgNPs and ammonia caused a color change from dark orange to amber, with a blue shift in absorption peak from 580 to 490 nm. Among various interferences, such as Fe^+3^, Cu^+2^, and Hg^+2^, AgNPs-based biosensors demonstrated significant selectivity toward ammonia. The recovery rates of ammonia from real samples were recorded between 98.8% to 102.5%.

Such findings collectively highlight the significant potential of plant-based synthesized AgNPs in biosensor development. Beyond offering unique physicochemical properties for various environmental and industrial applications, the green synthesis of AgNPs using plant sources further promotes eco-friendly approaches for these technologies.

### 4.6. Drug Delivery-Based Applications

The emergence of nanotechnology has significantly advanced the design of novel nanomaterials for therapeutic applications, including drug delivery systems with enhanced efficacy and biocompatibility [194]. Various types of nanostructures, such as nanofibers, lipid-based nanoparticles, polymeric carriers, and metallic nanoparticles, have been explored to improve drug loading, targeted delivery, and controlled release strategies [195,196,197]. AgNPs have become one of the most preferred nanomaterials for drug delivery applications due to their ease of functionalization, favorable stability under physiological conditions and antimicrobial characteristics. However, their wide range of utilization is hindered by certain drawbacks, including their toxicity potential. Accordingly, NPs synthesized via eco-friendly and cost-effective green synthesis methods, employing biological sources such as plant extracts and microorganisms (including bacteria, fungi and algae), has the potential to mitigate toxicity-related concerns and foster further research on AgNPs [25]. In particular, plant-mediated green synthesis methods are more extensively utilized by researchers due to their higher efficiency, lower biological risk, and the absence of a need for maintaining active cell cultures, unlike microorganism-based approaches. In addition, plant extracts (leaves, flower, fruit, callus, bark, peel, stem) have the potential to serve directly as natural reducing and capping agents due to their rich variety of bioactive compounds like carbohydrates, proteins, flavonoids, polyphenols, and enzymes. The stability of AgNPs is crucial for their performance in various applications, specifically in drug delivery applications. AgNPs with high stability can exhibit sustained drug release, enhancing their potential and bioavailability in drug delivery applications with a controlled release profile [198]. Moreover, since particle stability is directly related to the NP’s ability to interact with cells, unstable AgNPs may aggregate, leading to undesired targeting of other tissues. The ability to precisely control the stability, size, and surface properties of AgNPs through green synthesis further enhances their effectiveness as drug carriers, making them a promising option for future therapeutic applications. 

Accordingly, Gul et al. evaluated the efficiency of AgNPs green-synthesized using *Poa annua* for the delivery of the anticancer drug *Euphorbia dracunculoides* Lam. (EDL) [199]. The AgNPs, with an average diameter of 36.66 ± 7.85 nm and an oblate spheroid morphology, were loaded with EDL and coated with starch to form a novel drug delivery composite. The loading capacity and encapsulation efficiency of the nanocomposite were determined to be 82.5% and 85%, respectively. Furthermore, the researchers assessed the anticancer activity and biocompatibility of the composite under both in vitro and in vivo conditions. The DPPH scavenging effect was recorded as 81.00 ± 0.12%, and the total antioxidant capacity (TAC) was measured at 82.63 ± 0.07% at the highest tested concentration of 10.0 mg/mL. In vitro cytotoxicity studies demonstrated that at a concentration of 1000 µg/mL, the nanocomposite reduced the viability of RAW264.7 and SCC7 cells by approximately 20% and 30%, respectively, after 72 h of incubation. Under in vivo conditions, oral administration of nanocomposite at the highest dose of 300 mg/kg for 7 days in Sprague Dawley rats showed no significant cytotoxic effects, confirming the biocompatibility of the drug delivery system.

Ghobadi et al. synthesized AgNPs from different parts, including peel, flower, petals and calyx, of the *Punica granatum* L., commonly known as pomegranate. Spherical AgNPs ranging in size between 13.7 ± 0.4 nm to 18.2 ± 0.7 nm were coated onto amine-functionalized mesoporous silica NPs, then investigated for their ability to deliver anticancer drug DOX, which suffers from poor bioavailability, low selectivity and development of resistance as much of the anticancer drugs. DOX was loaded onto NPs at different ratios (1:1, 1:2, 1:5, 1:10), which then were tested following 24 h in terms of their loading capacity and loading efficiency. NPs at 1:1 ratio was utilized for further experiments due to their superior loading capacity percentage, 42.80 ± 0.10, in comparison to 21.70 ± 0.12, 8.78 ± 0.10, 7.53 ± 0.03 for 1:2, 1:5 and 1:10, respectively. The NPs achieved promising results, leading to 30% cell viability on MCF-7 cells treated with 100 μM of NPs. Additionally, they reduced HeLa cell viability to 50%, at a concentration of 10 μM, offering a novel perspective for anticancer therapy [200].

In another study, Altınay et al. developed a targeted drug delivery system consisting of AgNP-doped polyvinyl alcohol (PVA) hydrogel films loaded with ibuprofen. Researchers utilized *Equisetum telmateia* (horsetail) extracts for the green synthesis of AgNPs in order to enhance barrier properties to the delivery system. AgNPs/PVA films that were kept in 2 mL of ibuprofen solutions at a concentration of 1 mg/mL for 3 h of loading had drug loading efficiency of 67.5%. Further drug release tests revealed that the release profile depended on PVA content, with films containing 10% *w*/*v* PVA releasing 97% of the drug within 10 days, whereas films with 5% *w*/*v* PVA released 89% of the drug over 14 days. Authors also stated that incorporation of AgNPs enhanced the overall bactericidal efficiency of hydrogels, particularly against Gram-positive bacteria such as *S. aureus* [201].

Haripriya et al. used AgNPs derived from *Clitoria ternatea* extracts for the production of a delivery system. Researchers incorporated AgNPs into a mesoporous silica material (SBA-15) which was functionalized with 3-glycidoxypropyltrimethoxysilane (GPTMS) and tris(2-aminoethyl) amine (TAEA). Further, the resulting nanocomposite (SBA-15/GPTMS-TAEA-Ag) was loaded with Ciprofloxacin, achieving a loading capacity of 19.8% (*w*/*w*), to evaluate its drug release capability over varying time periods of 60, 95, 130, 760, 1260, and 1440 min. It was revealed that after 60 min drug release was slow, achieving only 4%. However, it increased steadily over time and reached up to 98.18% following 1440 min [202].

Even though the current literature on drug delivery systems utilizing plant-based green synthesized AgNPs remains limited when compared to their other applications, existing studies highlight their promising potential in this field. The ability of AgNPs to facilitate a more targeted release, improve stability and confer antimicrobial characteristics could further contribute to expanding their utilization in drug delivery systems.

All in all, plant-based AgNPs with diverse physicochemical properties have been extensively utilized for their significant biological activities. In this aspect, key physicochemical characteristics, such as size, morphology, and surface properties, are shown to be strongly influenced depending on the synthesis conditions and type of the plant extract, affecting the biological performance of AgNPs. The growing research background over the last years demonstrates the further potential of plant-based AgNPs, with varying characteristics, across various fields (Table 2).

## 5. Future Perspective and Toxicity

AgNPs have demonstrated significant potential in a variety of application areas, including antimicrobial therapy, agricultural treatments, biomedical applications, and environmental and industrial improvements [227]. Considering their growing literature background and novel strategies in these applications, AgNPs are expected to be utilized on a large basis. Despite the potential and experimental data supporting their applicability, certain important gaps must be addressed before such involvement.

Green-synthesis of NPs offers an environmentally friendly and safer alternative, compared to conventional methods. Among these, plant-based NP synthesis is one of the most widely preferred methods, specifically for AgNPs. Although plant-based synthesis of AgNPs exhibit cost-efficient and non-hazardous approach in the NP synthesis, the area is still suffering from some major challenges [228].

When comparing green synthesis to conventional approaches, the key aspects that make green synthesis a significant alternative are its eco-friendly nature and cost-effective methodology [229]. While conventional methods often require toxic chemicals and high energy consumption, the use of organic metabolites makes green synthesis a more environmentally sustainable approach by comparison. Still, it is important to highlight that, although plant-based green synthesis of NPs offers significant advances in controlling physicochemical properties and particle stability, it is not yet as well-established or widely applicable for industrial production as conventional methods. The current literature emphasizes the growing use of green synthesis for various applications and types of NPs production, but challenges remain in ensuring its reproducibility and consistency compared to conventional methods. There are several major factors that limit the utilization of green synthesis, including the selection of raw materials, the need for optimization of synthesis conditions (such as temperature and pH), the risk of contamination (if microorganisms are used as the source), challenges in scaling up production for industrial applications, and the determination of product quality and storage conditions [230]. It is also important to optimize the physicochemical properties of the synthesized NPs for specific applications. This requires analyzing the metabolites in plant extracts involved in nanoparticle synthesis, as well as understanding the synthesis parameters and reactants, since all of these factors influence the resulting properties [231]. Additionally, the lack of standardized protocols and the variability in plant extracts significantly hinder reproducibility, requiring further research to refine methods for large-scale production. This means that limitations related to NP quality, underdeveloped control over particle stability, and the variability of plant extracts due to growth and seasonal conditions should be prioritized for improvement. Addressing these issues is essential to minimize the risk of increased energy consumption, production costs, and reduced particle quality for applications in green synthesis [232].

**Figure 4:**
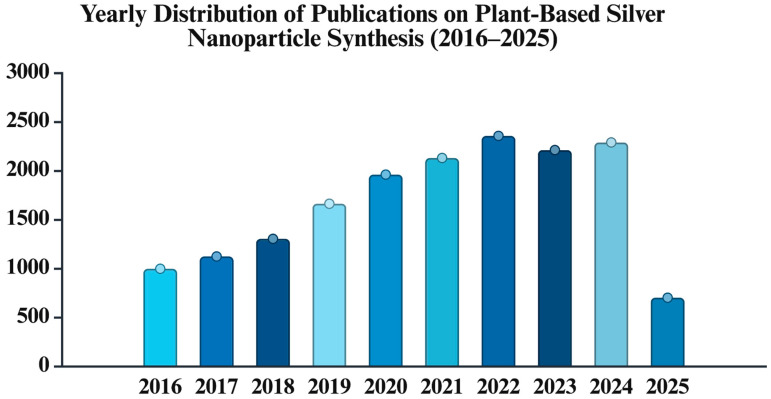
Yearly distribution of publications on plant-based silver nanoparticle synthesis (2016–2025, WoS, Topic Search). Data retrieved from Web of Science Core Collection using “Topic” search for “green synthesis of silver nanoparticles.” Publication counts represent the number of articles indexed per year from 2016 to 2025, with a total number of documents of 16,762 (Accessed on 12 May 2025).

The number of publications on plant-based AgNP synthesis has shown a consistent upward trend from 2016 to 2022, reflecting growing interest for green-synthesis in NP research (Figure 4). This highlights the expanding recognition of plant-mediated synthesis as a reliable, cost-effective and eco-friendly alternative to conventional methods. Even though there is a slight decline in the last two years (2023–2024), indicating a possible shift in focus to optimization-focused and application-based research, there is a considerable number of publications compared with the previous years. Regarding the sharp decrease observed in 2025, the figures are expected to reach the level of previous years once the missing data is completed by the end of 2025. The research focus on the plant-based synthesis of AgNPs will likely continue to grow, considering the key challenges, such as standardization of synthesis protocols and missing advancements in the scalability, will be addressed in the upcoming research.

Our analysis, as illustrated in Figure 4, aligns with recent reviews on the green synthesis of AgNPs [233,234,235], showing that plant-based methods for AgNP synthesis offer significant alternatives to traditional methods, with increasing research efforts. However, our approach presents the most recent data and includes an additional comparison of AgNPs with other metal nanoparticles in terms of green synthesis, as shown in Figure 1.

Figure 1 shows the overall trend in publications on the green synthesis of NPs over the last five years, covering a variety of metal-based NPs. In contrast, Figure 4 zooms in on the specific trend for plant-based synthesis of AgNPs over the past 10 years, illustrating the increasing focus on AgNPs. Together, these figures underscore the growing research emphasis on AgNPs in the field of green synthesis, highlighting both the general trend and the potential advancements in AgNPs research.

One such challenge is the variability of synthesis protocols. Factors such as the plant species used, the chemical composition of the extracts and methods for extraction overall contribute to inconsistencies in the size, morphology and biological activity of the NPs [236]. Despite the presence of many studies focusing on the optimization of these methods as we have discussed earlier, a lack of a universally accepted protocol that ensures consistent and reproducible results still remains. Addressing this, researchers have been developing statistical and experimental optimization strategies to minimize variability while improving the reproducibility [237,238].

Design of experiments (DoE) approaches such as the Box–Behnken and Plackett–Burman are among these strategies, which gained significant importance in current research. Using these approaches, researchers can systematically optimize various independent variables such as concentration of AgNO_3_, temperature, pH, incubation time and volume of the plant extracts used, to achieve NPs with desired size, shape, stability and yield [239]. These approaches minimize the number of experimental trials, hence, improve the overall efficiency of the process while also reducing the excess use of resources. By reducing the number of experiments needed in comparison to conventional single-parameter methods, DoE approaches not only save resources and time but also contribute to reproducibility and reliability in NP synthesis [240].

**Figure 5:**
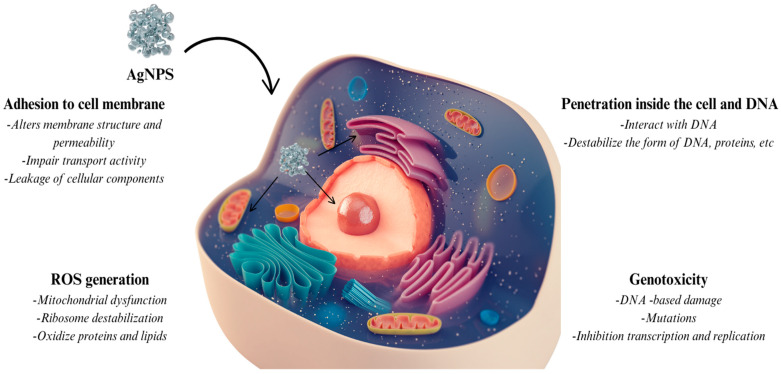
Mechanisms of AgNP toxicity [242,243].

Another challenge associated with AgNPs is their toxicity. Although green-synthesis approaches immensely minimize these toxic effects, there are still ongoing concerns about their dose-dependent cytotoxicity and long-term environmental impact especially at higher concentrations [241]. Specifically, through ROS generation, AgNPs can induce DNA damage, apoptosis and increase the levels of lipid peroxidation, affected by their physicochemical properties (Figure 5) [177].

The physicochemical properties of AgNPs offer a significant potential in their toxicity potential. Specifically, the size and surface charge of AgNPs directly influence their toxicity effects on human health. For instance, small-sized AgNPs tend to induce higher cytotoxicity compared to larger-sized particles, as a result of the increased release of Ag^+^ ions and their high surface area-to-mass ratio [244]. Similarly, particle stability plays an essential role in the toxicity capacity of AgNPs. In various types of applications, unstable AgNPs can aggregate, thus resulting in undesired accumulation on non-target tissues. Green synthesis offers a unique advantage in this context by utilizing biological agents, especially plant extracts, to control these properties. This approach not only ensures improved bioavailability but also allows for better control over the stability of the nanoparticles, further enhancing their efficacy and minimizing unwanted side effects in a variety of applications.

In a recent study focusing on this aspect, Tareq et al. illustrated that even though green-synthesized AgNPs had reduced toxic effects in comparison to chemical synthesis, adverse effects still occur at higher doses [245]. In their study, rats treated with AgNPs synthesized from *Psidium guajava* leaf extract at varying doses, 0.5, 5 and 10 mg/kg. It was revealed that AgNPs of 0.5 and 5 mg/kg showed negligible changes to control values. However, at the highest tested concentration of 10 mg/kg, AgNP administration led to a significant reduction in cortical monoamine neurotransmitters, serotonin (5-HT) and norepinephrine (NE), which is associated with many psychiatric diseases and mood disturbances such as depression. Similarly, Tarbali et al. evaluated dose-dependent toxic effects of AgNPs green synthesized from the leaves of *Myrtus communis* L. [246]. Following 21 days of exposure to AgNPs, rats administered 100, 200 and 400 ppm of AgNPs had anxieties and memory impairment, along with altered kidney, liver, spleen, and hippocampus redox status while the group received 50 ppm treatment not showing significant differences.

Considering the growing research background on plant-based AgNPs, future studies are expected to catalyze advancements across a range of scientific and industrial fields. The effect of plant biomolecules in the stability and reduction in AgNPs should be investigated in detail as well, to improve the current understanding of AgNPs’ toxicity and enhance their safe application in various fields.

One of the most highlighted areas of AgNP-based applications is biomedicine. In addition to their well-established antimicrobial activity, particularly in wound healing and agricultural applications, plant-based AgNPs show great potential in the development of drug delivery systems. By integrating these functionalities, AgNPs may improve therapeutic applications by enhancing treatment efficiency, reducing the side-effects and toxicity, and offering green-synthesized alternatives for cancer and inflammation-related disease management. Furthermore, their unique optical properties expand their utilization in industrial applications, enabling their incorporation into biosensors for the sensitive detection of a wide variety of biomolecules and environmental pollutants. Fostering interdisciplinary backgrounds with essential optimizations, AgNPs will lead scalable, safe, and commercially viable technologies.

## 6. Conclusions

Green synthesis has emerged as a promising approach to mitigate the hazardous potential and toxicity of AgNPs, to achieve maximum utilization of their biological properties. AgNPs are strong candidates in various fields, including but not limited to agricultural, antimicrobial, biomedical, industrial and environmental applications. Depending on certain conditions, specifically the physicochemical properties and administered doses, AgNPs are known with potent toxicity potential. In this context, green synthesis emerges as a safer, cost-effective and sustainable alternative in AgNP production. Recently, numerous plant species were used to synthesize NPs, in order to minimize adverse effects associated with silver and leverage the beneficial properties of phytochemicals found in the plant sources. However, while exploring new plant species for AgNP synthesis remains important, equal emphasis should be placed on optimizing existing, well-established protocols to ensure consistency, reproducibility and scalability in NP production. This approach will ensure the consistency of the growing research focus on the AgNPs, which will improve their safe and effective implementation across various fields, including biomedical, environmental and industrial applications.

## Figures and Tables

**Table 2:** Physicochemical properties and biological applications of plant-based AgNPs from recently conducted studies.

Plant Source	Application Type	Biological Activities	Physicochemical Property of the AgNPs	References
*Lallemantia royleana* leaf extract	Antimicrobial Biomedical Photocatalytic	Antibacterial activity against multiple bacteria species Antifungal activity against *Candida glabrata* and *Candida albicans* Degradation of methylene blue Anti-inflammatory activity Antioxidant activity	Spherical morphology Average size of 34.5 ± 1.6 nm SPR peak at 425 nm Zeta potential of −24.1 mV	[83]
*Mangifera indica* Aqueous Leaf Extract	Antimicrobial Agricultural	Antibacterial activity against MDR bacteria species Enhanced biochemical constituents of infected wheat crops	Spherical morphology Average size of 52.8 nm SPR peak at 475 nm	[203]
*Azadirachta indica* leaf extract	Antimicrobial Agriculture	Antifungal activity against *Alternaria solani* Increased resistance of susceptible tomatoes	Spherical morphology Particle size between 22 and 30 nm SPR peak at 424 nm	[204]
Aqueous leaf extract of *Perilla frutescens* L.	Antimicrobial Biomedical	Antibacterial activity against *E. coli* and *S. aureus* Antifungal activity against *Candida albicans* Toxicity against MCF-7 cancer cells Antioxidant activity	Spherical morphology Average size of <61 nm SPR peak at 436 nm Zeta potential of −18.1 ± 0.72 mV	[60]
Leaf Extract of *Carissa carandas* L.	Antimicrobial Biomedical	Antibacterial activity against various human pathogenic bacteria Antioxidant activity	Average size of 35 ± 2 nm SPR peak at 432 nm and 444 nm	[205]
Green banana pulp extract	Antimicrobial Industrial	Antibacterial activity against *E. coli* and *S. epidermidis* Increased voltage, current and voltage regulation of electrochemical cells	Spherical morphology Average size of 42.97 nm SPR peak at 475 nm	[206]
*Hypericum perforatum* L. aqueous extract	Antimicrobial Agricultural	Antipathogenic activity against several food pathogens Reduction in bacterial growth rate	Spherical and monodisperse morphology Average size between 20 and 40 nm SPR peak at 425 nm Zeta potential of −19 mv	[207]
*Ferula gummosa* Boiss. gum extract	Antimicrobial Biomedical	Antibacterial activity against several bacteria strains Increased antioxidant activity with chitosan coating Notable anticancer activity against cell lines	Spherical morphology Average particle size of 5.63 nm Zeta potential of 52.2 mV SPR peak at 420 nm	[208]
*Sida schimperiana Hochst. ex A. Rich* leaves extract.	Antimicrobial	Antibacterial activity against several bacteria strains	Face-centered cubic morphology Average size of 26.27 nm	[209]
*Senna siamea*	Agricultural	Increased shoot and root length, seed oil content, seed yield, number of branches, photosynthetic pigments and biochemical features of *Trachyspermum ammi* (L.) inoculated with *Meloidogyne incognita*	Round and polydiverse morphology Size ranging from 5 to 60 nm SPR peak at 430 nm	[210]
*Coffea arabica* husk extract	Antimicrobial Biomedical	Antibacterial activity against several bacteria strains (*E. coli*, *S. aureus*, *P. aeruginosa* and *B. subtilis*) Selective cytotoxicity towards MCF-7 cancer cells	Spherical morphology Average size of 147 nm Zeta potential of −27.8 mV	[211]
Taro corm extract	Biomedical Antimicrobial	Antibacterial activity against various pathogenic bacteria Antioxidant activity In vivo (rabbits) wound healing activity	Spherical morphology Average size of 244.9–272.2 nm SPR peaks at a range of 438–445 nm Zeta potential of −18.8 mV	[212]
*Rhodiola rosea*	Antimicrobial Biomedical	Antibacterial activity against *S. aureus* and *P. aeruginosa* Antioxidant activity Increased IL-10 levels and decreased pro-inflammatory cytokine levels in murine model burn wound healing	Spherical morphology Size of 20 nm SPR peak at 430 nm Zeta potential of −68.38 ± 3.4 mV	[173]
*Rhizophora apiculata*	Antimicrobial Biomedical	In vitro anti-inflammatory activity by 71.65 ± 0.88% Notable anticancer activity In vitro wound healing activity by 82.79% Antioxidant activity	Irregular morphology Size between 35 and 100 SPR peak at 459 nm Zeta potential of −6 mV	[213]
Mulberry leaf	Antimicrobial Biomedical	Antioxidant activity Antibacterial activity against *E. coli* and *S. aureus* In vitro anti-inflammatory activity In vitro antidiabetic activity by 47.03% amylase inhibition Anticancer activity with cell viability of 17.28%.	Spherical morphology Average size of 100 nm SPR peaks in the range of 420–450 nm Absolute zeta potential of >30 mV	[214]
*Brachychiton populneus* Leaf Extract	Antimicrobial Biomedical	Antioxidant activity In vitro antidiabetic activity by 80% amylase inhibition In vitro anti-inflammatory activity by protein denaturation inhibition	Cubical morphology Average size of 15 nm SPR peak at 453 nm	[215]
*Euphorbia hirta*	Antimicrobial Industrial	Antibacterial activity against *E. coli* and *S. aureus* Antifungal activity against *Candida albicans* and *Aspergillus niger* Observable color change with heavy metal solutions	Hexagonal morphology Average size of 30 nm SPR peak at 470 nm	[216]
*Elettaria cardamomum* Seed Extract	Antimicrobial Biomedical	Antibacterial activity against *E. coli* and *S. aureus* Antioxidant activity Anticancer activity with cytotoxicity of 83.66%	Spherical morphology Average size of 19 nm SPR peak at 412 nm	[217]
*Allium cepa* L. leaves extract	Antimicrobial Environmental	Antibacterial activity against several bacteria strains Antifungal activity against several fungal strains Electrochemical nitrite detection	Spherical morphology Size ranging 1 to 30 nm SPR peak at 432 nm	[218]
*Ocimum sanctum* (tulsi)	Biomedical	Antibacterial activity against *E. coli* In vitro scratch wound closure rate by 45% Improved mechanical properties of film	Spherical morphology Average size of 28.95 ± 7.74 nm Zeta potential of −17.8 mV	[171]
*Boerhavia diffusa* extract	Biomedical	Antioxidant activity Concentration-dependent anticancer activity by apoptosis induction	Spherical morphology Average size of 34 nm SPR peak at 427 nm	[219]
*Cymbopogon jwarancusa*	Agricultural Environmental	Increased seed germination of wastewater treated maize seedlings up to 60% Increased root and shoot lengths	Spherical and square-to-rectangular morphology Average size of 31 nm SPR peak at 433 nm	[220]
Tea tree leaves extract	Agricultural	Antibacterial activity against several bacteria strains Antifungal activity against several fungal strains Microbicidal activity on parchment samples Increased mechanical property of parchment samples	Spherical, oval, and hexagonal morphology Size ranging 20 to 50 nm SPR peak at 468 nm	[221]
*Lavandula angustifolia* Mill	Biomedical	Antiulcerogenic activity against ethanol-induced gastric ulcers in rats Antioxidant activity	Spherical and cubical morphology Average size of 69.35 nm SPR peak at 420 nm	[222]
*Malachra alceifolia* leaf extract	Environmental Biomedical	Antibacterial activity against *S. aureus* and *P. aeruginosa* Antioxidant activity up to 53% scavenging ability Photocatalytic activity against methylene blue under direct solar radiation	Spherical morphology Average size of 28 nm SPR peak at 455 nm	[223]
*Trigonella foenum-graecum* Seeds	Environmental	%94.5 degradation of crystal violet dye under UV irradiation Increased water quality parameters of wastewater Antibacterial activity against *E. coli* and *S. aureus*	Spherical morphology Average size of 20–50 nm SPR peak at 439.29 nm	[224]
*Helichrysum graveolens*	Environmental	Antibacterial activity against several bacteria strains Photocatalytic activity against methyl orange	Spherical morphology Average size of 11 nm SPR peak at 439 nm	[225]
*Acacia raddiana* leaves	Industrial	Observable detection of multiple heavy metals Heavy metal sensing (Pb, Cu and Co) within real wastewater samples ranging 42.33% to 100.72%	Spherical morphology Average size of 77.35 ± 50.4 nm SPR peak at 423 nm Zeta potential of − 32.2 mV	[82]
Phulae pineapple peel extract	Industrial	Detection of serum albumin concentrations from 10 to 400 μg/mL	Spherical morphology Size ranging 10 to 30 nm SPR peaks ranging from 440 to 460 nm	[226]

## Data Availability

No new data were created or analyzed in this study.
